# Study of grapevine endophytes’ interaction with the host and their potential as biocontrol agents as sustainable alternative to agrochemicals

**DOI:** 10.3389/fmicb.2026.1758446

**Published:** 2026-02-18

**Authors:** Simona Pizzi, Alessandra Di Canito, Elena Palencia Mulero, Carmen Cris De Oliveira Nobre Bezerra, Valentina Ricciardi, Giuliana Maddalena, Evelyn Maluleke, Roberto Carmine Foschino, Silvia Laura Toffolatti, Daniela Fracassetti, Gabriella De Lorenzis, Gustavo Cordero-Bueso, Mathabatha Evodia Setati, Ileana Vigentini

**Affiliations:** 1 Department of Biomedical, Surgical and Dental Sciences (DiSBIOC), Università degli Studi di Milano, Milan, Italy; 2 Department of Agricultural and Environmental Sciences (DiSAA), Università degli Studi di Milano, Milan, Italy; 3 South African Grape and Wine Research Institute, Stellenbosch University, Stellenbosch, South Africa; 4 Department of Food, Environmental and Nutritional Sciences (DeFENS), Università degli Studi di Milano, Milan, Italy; 5 Department of Biomedicine, Biotechnology and Public Health, Faculty of Sciences, University of Cádiz, Puerto Real, Spain

**Keywords:** *Aureobasidium pullulans*, *Bacillus velezensis*, *Botrytis cinerea*, elicitation, host interaction, sustainability

## Abstract

In sustainable vineyard management, biocontrol agents (BCAs) offer a promising alternative to chemical pesticides. This study focuses on characterizing endophytes isolated from grapevines in different regions of northern Italy for (i) their effectiveness against *Botrytis cinerea* through *in vitro* tests and (ii) their capability to survive in the presence of copper and other commercial fungicides with the final goal of investigating their possible future application for an integrated approach in vineyard. *Aureobasidium pullulans* strains ED203, ED206, ED217, and ED221 demonstrated significant antagonistic activity toward *B. cinerea*, while *Bacillus velezensis* ED163 showed the highest levels of pathogen inhibition. Volatile organic compounds (VOCs) analysis identified three distinct microbial clusters based on BCAs volatile profiles, highlighting the production of antifungal compounds such as alcohols, esters, and terpenes to counteract the pathogen growth. The investigated BCAs showed tolerance to high concentrations of copper (up to 100 mg/L) and the fungicide SWITCH (1 g/L), but they were sensitive to a standard oenological dose of sulfur dioxide (50 mg/L). Finally, *in vivo* tests on grapevine leaves confirmed the ability of *B. velezensis* and *A. pullulans* strains ED203 and ED221 candidates to effectively reduce the mycelium infection on plant tissue standing out as strong candidates for pest management. The interaction between BCAs with the host was confirmed through leaves cell culture use, highlighting the capability of the best candidate selected in the study to modulate plant response in presence of pathogen.

## 1 Introduction

The use of pesticides in agriculture is due to the need to prevent crop diseases and, as a consequence, to potentially increase productivity. However, excessive use can lead to serious damage to the environment and human health (Compant et al., 2011). In viticulture, the problem with pesticide application is the formation of residues that may influence the quality of grapes and wine. Moreover, the continuous consumption of contaminated products may result in health issues for consumers, so regulating their use is necessary. To address this, the European Commission introduced EC Directive No 128/2009 to promote the sustainable use of pesticides and to reduce both their application and negative impact. This directive has recently been revised within the framework of the “Farm to Fork” strategy, which aims to further reduce the use of chemical pesticides and promote more sustainable agricultural practices (Pizzi et al., 2025).

In addition to the synthetic fungicides commonly applied in viticulture, copper-based products, originally introduced as “Bordeaux mixture” (Koledenkova et al., 2022), are still widely used for the control of downy mildew (Mackie et al., 2012). However, copper is a non-degradable heavy metal, and its repeated use has led to its accumulation in the topsoil, often reaching toxic levels, causing plant stress and reducing soil fertility (Mengel et al., 2001). Due to these environmental concerns, the European Union has limited copper usage to 28 kg per ha over a period of 7 years (as of 2021), with typical application rates ranging from 200 to 400 g Cu ha^–1^ (20–40 mg Cu m^2^), which can reduce infections by at least 90% (Dumitriu et al., 2022). The final objective is to phase out copper altogether, as it is currently listed among the EU’s candidates for substitution (Tamm et al., 2022).

Protective treatments are not limited to the vineyard stage; they are also applied during winemaking. The most common additive is sulfur dioxide (SO_2_), which is used to prevent oxidation, microbial spoilage, undesirable secondary fermentations, and the proliferation of *Brettanomyces*, thereby preserving wine quality (Santos et al., 2012). However, excessive SO_2_ can negatively affect the sensory properties of the final product and cause health issues, leading the International Organization of Vine and Wine (OIV) to regulate the maximum authorized concentration in wines to 150 mg/L for red wines and 200 mg/L for white wines (Commission Regulation (EC) No 607/2009, 2009).

To reduce the use of agrochemicals, microorganisms that naturally reside within plant tissues (i.e., endophytes) could represent an innovative protection strategy against fungal pathogens as biological control agents (BCAs), provided that they exhibit antagonistic activity and tolerance to commercial chemical products such as fungicides and copper (Morales-Cedeño et al., 2021).

Additionally, it is important to ensure that the use of SO_2_ in pre-fermentation winemaking phases can remove the BCAs presence to avoid subsequent fermentation issues.

Many endophytes isolated from grapevines can counteract the development of pathogens through a variety of mechanisms. These include nutrient competition, the production of lytic enzymes, such as chitinases, cellulases, proteases, and β-1,3-glucanases, involved in fungal cell wall degradation (Morales-Cedeño et al., 2021), and biofilm formation as a spatial competition strategy to colonize grape surfaces and promote adherence and proliferation (Cordero-Bueso et al., 2017; Di Canito et al., 2021). Furthermore, endophytes produce secondary metabolites such as phytoalexins, biocide compounds, and cyanhydric acid (HCN) as well as antibiotics, and volatile organic compounds (VOCs). These metabolites can exert an inhibitory effect on phytopathogens limiting their growth, sending signaling defense and gene regulation (Samad et al., 2017). The latter mode of action includes the release of terpenes that show a broad range of the biological properties, including cancer chemopreventive, antimicrobial, antifungal, antiviral and antiparasitic properties (Chen et al., 2021). Other VOCs such as esters and alcohols have also demonstrated inhibitory effects on fungal mycelium growth (Maluleke et al., 2022).

The use of endophytes as BCAs, coupled with environmentally sustainable practices, can be useful to counteract the proliferation of pathogenic fungi such as *Botrytis cinerea*, the causal agent of gray mold, one of the major diseases that infect multiple grape organs including leaves, green shoots, rachides, flowers, bunch trash and berries (Elad et al., 2007).

BCAs characterization based on their ability to inhibit specific fungal pathogens is crucial to determine their potential integration with conventional chemical treatments currently employed in vineyards. Theoretically, BCAs application would consist solely of inoculating selected microorganisms capable of effectively limiting pathogen infection. However, current research has not confirmed yet that it is possible to achieve results in vineyards comparable to those obtained with traditional chemical treatments. Therefore, investigating integrated biological and chemical strategies remains a valuable approach to prevent grapevine diseases while reducing pesticide use.

It is important to note that *in vitro* antagonistic trials are useful to focus on isolated mechanisms of interaction between endophytes and pathogens, but they do not fully capture the complexity of vineyard ecosystems. For this reason, these trials are usually coupled with *in vivo* tests that involve the interactions of antagonists with the host under controlled conditions, which allow for the evaluation of interactions between antagonists and the host plant in a more realistic context.

This study aimed at selecting grapevine-associated endophytes as potential biocontrol agents against *B. cinerea*. Their antagonistic activity was assessed through *in vitro* assays, analysis of volatile organic compound production, and *in vivo* tests on grapevine leaves. Their tolerance to common vineyard fungicides, copper, and oenological SO2 was evaluated to determine their suitability for integrated disease management and winemaking applications. Finally, the interaction with grapevine cell cultures with most promising isolate *via* phenylpropanoid-related gene expression was further examined to investigate how BCAs influence the plant’s defensive response in the presence of a pathogen, providing insights into the regulatory mechanisms that BCAs may trigger.

## 2 Materials and methods

### 2.1 Selection of BCA candidates

The collection of grapevine material, endophytes isolation procedures and NCBI GenBank corresponding accession numbers were described and reported in Pizzi et al. (2025). All sequences were deposited in the NCBI GenBank databank, and the corresponding accession numbers are reported in the same article. Endophytes strains from different origins against *B. cinerea* strain B05.10 were tested for their potential antagonistic activity (Supplementary Table 1).

Briefly, fresh cultures of bacteria were obtained in Luria-Bertani (LB) broth (Scharlau, Barcelona, Spain), while yeasts were cultivated in Potato Dextrose Broth (PDB) (Scharlau, Barcelona, Spain), both at 25°C for 24h. Then, cell concentration was estimated by optical density (OD) measurements; for bacterial cultures, OD readings at 600nm were adjusted to 10^7^ CFU/mL using the following approximations OD_600_ = 0.2 ≈ 10^7^ CFU/mL for *Bacillus* spp. (Biesta-Peters et al., 2010), and OD_600_ = 0.1 ≈ 5 × 10^7^ CFU/mL for other genera (Gu et al., 2023). Yeast cultures were quantified by OD at 660nm, with OD660 = 0.1 corresponding to approximately 106 CFU/mL (Guthrie and Fink, 1991).

These culture media, growth conditions, and cell concentration estimation were consistently applied across all subsequent experiments.

In order to evaluate the antifungal activity, pathogenic fungus strain *B. cinerea* B05.10 (Colección Española de Cultivos Tipo, Burjassot, Valencia, Spain) was used. Spore suspension was obtained by scratching and filtering with a double layer of sterile gauze the mycelium from a 7-day-old culture inoculated on PDA (Potato Dextrose Agar) medium (Scharlau, Barcelona, Spain) using 10 mL of sterile water. After that, spores were centrifuged at 6,080 *g* × 20 min (Hettich, ROTINA 380R, Tuttlingen, Germany) and the pellet was washed with 5 mL sterile distilled water. Conidial concentration was determined using a Neubauer-Improved Double Chamber (Paul Marienfeld GmbH & Co. KG, Lauda-Königshofen, Germany) and adjusted to 10^6^ spores/mL. Spore suspensions were stored at −80°C in 50% (v/v) glycerol.

### 2.2 Dual-culture and double-Petri dish assays

To preliminary evaluate the antagonistic activity against fungal pathogens, the isolates were subjected to *in vitro* tests using the dual-culture (DC) plate (Supplementary Figure 1A). Ten μL aliquots of the microbial suspension (10^5^ and 10^4^ cells for bacteria and yeasts, respectively) were spotted 1 cm from the edge of a Petri dish containing PDA. The plate was then inclined to allow the cell suspension to spread across the surface, forming a continuous line approximately to about 1 cm from the opposite edge of the plate. Subsequently, 10 μL of a mold spore suspension (containing 10^4^ spores) were inoculated approximately 2 cm from the edge of the plate and air-dried under sterile conditions. Plates were incubated at 25°C and daily monitored for mycelial growth up to 7 days, following the protocol described by Fernandez-San et al. (2021). Plates inoculated only with the fungal pathogen were used as control. All assays were performed in triplicate.

Isolates showing inhibitory activity in the DC assay < 40%, corresponding to a clearly visible reduction in mycelial expansion, were further tested in the DPD assay (Supplementary Figure 1B), as described by Di Francesco et al. with slight modifications (Di Francesco et al., 2015). This method, which prevents physical contact between the organisms, was employed to assess whether the inhibition of conidial germination was due to VOCs released by the BCAs. PDA plate center was inoculated with 10 μL of spore suspension, while another PDA plate with 10 μL of BCA suspension. After drying in a laminar flow hood, the two base plates were sealed together with double layer Parafilm^(R)^ and incubated at 25° C for 7 days. Control plates were inoculated only with the fungal pathogen. The experiment was conducted in triplicate. Pathogen mycelial growth was quantified for both assays using SketchAndCalc software (Dobbs, 20 February 2011), and the inhibition rate (%) was calculated as: Inhibition (%) = [(AC – AA)/AC] × 100 (Ruiz-Moyano et al., 2016), where AC is the area of the fungal colony in the control and AA is the area in the presence of the antagonist.

### 2.3 Tolerance tests

#### 2.3.1 Resistance to copper and commercial fungicide

The tolerance of previously selected BCAs to chemical stressors was evaluated using both commercial fungicide and copper. For fungicide assays, SWITCH (Syngenta), containing 37.5% Cyprodinil and 25% Fludioxonil, was tested at 0, 0.5 and 1 g/L, with a medium containing no fungicide used as control. For copper tests, different concentrations of Cu^2+^, prepared from a 0.2 M stock solution of CuSO_4_⋅5H2O sterilized by 0.2 μm filtration were applied. The concentrations of Cu^2+^ ranged from 20 to 900 mg/L, corresponding to 79–3543 mg/L of CuSO_4_⋅5H2O (0.32–14,19 mM). Specifically, the concentrations tested were: 20, 64, 100, 200, 300, 400, 500, 600, 700, 800, and 900 mg/L Cu^2+^, with a medium containing no copper used as control.

Cell suspensions in PDB medium supplemented with the different fungicide or copper concentrations were prepared at 106 CFU/mL for yeasts and 10^7^ CFU/mL for bacteria, and 6-well plates (Thermo Scientific™ Nunc™ Cell-Culture Treated). Multi-well plates were incubated statically at 25°C for 7 days. BCA tolerance was assessed visually, with sensitive BCAs defined as those unable to grow compared to control wells without fungicide or copper. All assays were performed in triplicate.

#### 2.3.2 Resistance to sulfur dioxide

The resistance of BCAs to sulfur dioxide (SO2) was assessed following the protocol outlined by the OIV (RESOLUTION OIV-OENO 370-2012), with slight modifications. Initially, 10% stock solution of SO_2_ was prepared using the potassium metabisulfite dissolving in a distilled water and sterilized by filtration through 0.2 μm filters. For the assay, 200 μL of BCA suspension (10^6^ cell/mL) were inoculated into 10 mL of liquid PDA medium adjusted to pH 3.5 and supplemented with 50 mg/L of SO_2_. Cultures were incubated at 25°C for 4 days. PDB medium without SO_2_ served as a control. The experiment was conducted in triplicate. Cellular growth was evaluated both by OD measurement (600 nm for bacteria, 660 nm for yeasts) and by viable cell counts on PDA plates at the end of the test.

#### 2.3.3 Yeast molecular typing

The molecular characterization of *A. pullulans* candidates was obtained by intron splice site PCR amplification (ISS-PCR) and rep-PCR fingerprinting. Yeasts were grown overnight at 25°C in liquid YPD medium with shaking and DNA extraction was performed. ISS-PCR amplification was carried out using primers EI1 (5′-CTGGCTTGGTGTATGT-3′) and LA2 (5′-CGTGCAGGTGTTAGTA-3′). For 25 μL of PCR reaction, 80–100 ng of genomic DNA was added to a Wonder Taq Thermostable DNA polymerase reaction mix (EuroClone, Italy) consisting in 5 μL of Wonder Taq Reaction buffer [1X], Wonder Taq [2.5 U/μL], Forward and Reverse Primers [0.75 μM] and molecular grade water. Amplification was carried on in a Mastercycler nexus (Eppendorf, Hamburg, Germany) following the cycle described by Vigentini et al. (2011), with an initial denaturation to 94°C for 3 min; 40 amplification cycles with 1 min at 94°C; 2 min at 46°C; and 1 min at 72°C. The final cycle was followed by an additional 5 min at 72°C.

For rep-PCR fingerprinting, the single oligonucleotide primer (GTG)5 (5′-GTGGTGGTGGTGGTG-3′) was used. PCR reactions were prepared as described above. The cycling conditions were as follows: initial denaturation at 94°C for 1 min; 35 cycles of 30 s at 95°C, 15 s at 53°C, and 30 s at 72°C; and a final extension at 72°C for 5 min. Negative and positive controls were included in all runs. PCR products were separated by electrophoresis on 1.5% (w/v) agarose gels supplemented with 0.5 μg/mL ethidium bromide in 1 × TAE buffer (40 mM Tris–acetate, 1 mM EDTA, pH 8.0) and run at 100 V for 45 min. DNA banding patterns were visualized using a GelDoc UV transilluminator (Bio-Rad, Hercules, CA, United States).

### 2.4 Production of volatile organic compounds

Volatile compound production resulting from the interaction between selected BCAs and *B. cinerea* was evaluated by Solid Phase Microextraction (SPME) coupled with gas chromatography–mass spectrometry (GC–MS), using the protocol described by Fracassetti et al. with some modifications (Fracassetti et al., 2020). A dual-medium setup was prepared in 20 mL glass vials, with 3 mL of Potato Dextrose Agar (PDA) on one side for *B. cinerea* growth, and 3 mL of Grape Juice Medium (GJM; 50% grape juice, 50% distilled water, 2% agar) on the opposite side for BCA inoculation. *B. cinerea* strain B05.10 was streaked on the PDA portion and, after 24 h, BCAs were inoculated on the GJM side. Vials were incubated at 25°C under light conditions for 7 days. The SPME was carried out by carboxen-polydimethylsiloxane-divinylbenzene (CARPDMS-DVB; 50/30 μm × 1 cm) fiber (Supelco, Bellefonte, PA, United States), connected with an autosampler (HTA autosampler, Brescia, Italy), at the following conditions: incubation for 10 min at 50°C without agitation; extraction for 30 min at 50°C; and desorption for 20 min. The separation was achieved by a ZB-WAXplus column (30 m × 0.250 mm × 0.25 μm) (Phenomenex, Torrance, CA, United States) using helium as a carrier gas at 1 mL/min flow rate. The oven temperature was initially set at 40°C and held for 5 min, ramped at 1.5 °C/min up to 220°C and held for 10 min. The transfer line temperature was set at 230°C and the source temperature was set at 250°C. The mass spectrometer operated in electron ionization mode at 70 eV using the full scan mode. The MS detector registered the m/z in the range from 33 Da up to 350 Da. The ions used for identification of target molecules were chosen according to the National Institute of Standards and Technology (NIST) MS Search 2.0 library fixing a fitting value (R) of minimum 90%. VOCs were quantified as relative signal intensities (peak areas) and used for comparative profiling, referring to VOCs produced by BCA alone and in the presence of *B. cinerea* as well as by *B. cinerea* alone.

### 2.5 *In vivo* assay for inhibitory activity

The candidates selected through *in vitro* antagonism assays were further evaluated *in vivo*, to assess the interaction with grapevine leaf tissue. Approximately twenty healthy grapevine leaves of cv. Merlot were collected from greenhouse-grown plants located in the Department of Agricultural and Environmental Sciences collection (University of Milan, Milan, Italy). Leaves were cut into uniform discs (Ø 2.5 cm) using a cork borer and surface-sterilized by sequential immersion in 0.5% sodium hypochlorite, 70% ethanol, and three rinses with sterile distilled water. Sterilized discs were then submerged for 5 min in a suspension of each BCA (106 cells/mL), previously cultured in 5 mL of YPD broth at 25°C for 48 h, or in sterile distilled water (negative control). After treatment, discs were incubated overnight at 25°C. The following day, infection was initiated by placing an agar plug containing *B. cinerea* mycelium (strain B05.10) in the center of each disc. Samples were incubated at 25°C, and disease progression was assessed for 7 days post-inoculation. Relative damage was assessed by quantifying the necrotic area on each leaf disc using ImageJ. For every sample, the total disc area and necrotic area was measured to calculate the percentage of tissue damage. The mean damage observed in the *B. cinerea* (positive control) was set as the 100% reference, and the damage values of all treatments were expressed relative to this baseline. Statistical analysis was performed using one sample *t*-test. All treatments were performed in 3 biological replicates, each consisting of one discs obtained from a different leaf.

### 2.6 Evaluation of BCA/plant/pathogen interaction in plant cell culture experiments

#### 2.6.1 Callus preparation

Calli from the *in situ* wild-growing *Vitis vinifera* subsp. *Sylvestris* (44.973866, 9.2224803; Montalto, Piedmont, Italy), hereafter referred to simply as *V. sylvestris*, were prepared as described. Briefly, young stems (7–8 cm long) were excised from plants at the flowering state and immediately transported to the laboratory. For callus induction, the first four expanded shoot leaves were selected. Leaves were first rinsed under tap water for 10 min and then surface-sterilized by sequential immersion in 90% ethanol for 1 min, 2.5% sodium hypochlorite for 10 min, and sterile water for two 10-min washes. Subsequently, small leaf explants (0.5 × 0.5 mm) were excised and cultured on 9-cm Petri dishes containing solid full-strength Murashige and Skoog (MS) medium (Duchefa Biochemie, Netherlands), supplemented with 30 g/L sucrose (Duchefa Biochemie), 1 mg/L 6-Benzylaminopurine (BAP) (Sigma-Aldrich, Germany), and 0.1 mg/L 2,4-Dichlorophenoxyacetic acid (2,4-D) (Sigma-Aldrich). The medium was solidified with 7 g/L agar (Duchefa Biochemie) and adjusted to pH 5.8 prior to autoclaving at 121 °C for 20 min. Cultures were maintained in darkness at 24 ± 1 °C to induce callus formation. Once callus tissue had developed (approximately 2 months after culture initiation), cultures were maintained under the same conditions and sub-cultured onto fresh solid medium every 2 months.

#### 2.6.2 Plant cell culture tests

Elicitation experiments in liquid cell cultures were conducted using the most effective BCA identified in antagonistic assays. To establish the liquid co-cultures, 2.0 g of fresh callus tissue was inoculated in 100 mL of MS liquid medium, supplemented as described for callus induction, and incubated at 26°C in darkness. After 7 days, the subcultures were performed at a 1:10 ratio and elicitation treatments with the BCA were initiated 4 days later. Briefly, the BCA was grown in Luria–Bertani (LB) broth at 25°C for 24 h. Spores of two *B. cinerea* strains, B05.10 and UCA992 (Colección de Levaduras y Hongos de la Universidad de Cádiz, Spain), were harvested from freshly grown mycelium on agar plates and quantified as previously described. For co-cultivation, 100 μL of the BCA suspension (10^7^ cells/mL) and 100 μL of *B. cinerea* spore suspension (106 spores/mL) were added simultaneously to the liquid callus medium and incubated at 25°C for 8 days. Plant cell cultures without inoculation were served as controls. The elicited co-cultures were subsequently used for electron microscopy analysis of microbial interactions and for evaluating the expression of genes involved in the phenylpropanoid pathway.

#### 2.6.3 Chemical fixation and plant cell dying by SEM microscopy

Cells from liquid co-cultures were collected after 8 days, to evaluate the interaction between cells during the experiments through electronic microscopy, as described by Minuti et al., with some modifications (Minuti et al., 2023). The preparation of the sample involves fixation; it must be dried under controlled temperature and pressure conditions (use of the dryer at the critical point) and, therefore, mounted and covered with conductive material (sputter coating).

Ten milliliter of co-culture were centrifuged at 6,080 *g* × 20 min (Hettich, ROTINA 380R, Tuttlingen, Germany) and resuspended in 5 mL of sterile water. Then, 100 μL of cells were fixed on a cover glass covered with L-polylisine solutions 0.1% (Sigma-Aldrich, St. Louis, MO, United States) and then in 10 mL glutaraldehyde 2.5% and cacodylate 0.1M under the laminar hood at 25 °C for 2 h. The fixative solution was then removed, and samples were washed three times for 15 min each in 0.1 M cacodylate buffer. Subsequent dehydration was performed by sequential immersion in acetone solutions at 70, 90, and 100% concentrations for 30 min each. Critical point drying was carried out using a Balzers CPD 030 apparatus. Samples were immersed in liquid CO_2_, which was brought to its critical point (31°C, 72 bar) to achieve drying without surface tension artifacts. Dried samples were mounted on 12 mm aluminum stubs using carbon bi-adhesive tape and sputter-coated with a thin gold layer using a Cressington 208 HR sputter coater to enhance conductivity.

SEM imaging was conducted at the Central Laboratory of the University of Cádiz using a Scios 2 DualBeam SEM (Thermo Scientific). Image acquisition and 3D reconstruction were performed with Auto Slice & View 4 (AS&V4) and Avizo software (Thermo Scientific).

#### 2.6.4 Phenylpropanoid pathway expression

Gene expression analysis was performed using samples from liquid co-cultures collected at 4 and 8 days after BCA elicitation. A non-inoculated cell culture suspension, aged 11 days, was used as the reference sample. Total RNA was extracted using the RNeasy Plus Minikit (Qiagen, Hilden, Germany) and RNA concentration was quantified using Qubit 4 Fluorometer (ThermoFisher). cDNA synthesis was performed using Oligo dT Primer (50 μM) primers and the PrimeScript™ RT reagent Kit (Perfect Real Time; Takara Bio Inc.). Quantitative real-time PCR (qRT-PCR) was carried out on a CFX Connect Real-Time PCR Detection System (Bio-Rad) using iTaq Universal SYBR Green Supermix (Thermo Fisher Scientific, Waltham, Massachusetts, United States). Specific primers (*STS1, VvABCG44*, and *EFα1*) (metaBIOn, Germany) were used for amplification (Pietrowska-Borek et al., 2021). The genes analyzed were selected based on *V. vinifera* sequences, given the extensive availability of reference data in the literature. The gene sequences were confirmed by targeted sequencing in *V. sylvestris* and then have been deposited in GeneBank with the accession code PX559946-PX559947. Relative gene expression was calculated using the comparative CT (2^–ΔΔCT^) method, with *EFα1* serving as the endogenous control (Duan et al., 2015). Primer sequences and GenBank accession numbers are listed in Table 1.

**TABLE 1:** Primer sequences used for quantitative real-time polymerase chain reaction (Pietrowska-Borek et al., 2021).

Gene	Gene bank ID	Forward primer (5′–3′)	Reverse primer (5′–3′)	Amplicon length (bp)
*STS1*	XM_002264419.2	CGCCAGGAGATAATCACTGCT	GCACCAGGCATTTCTACACC	134
*VvABCG44*	AB910387.1	TAGGAGTGGTTGCAGCTGTG	TTTTGCTCCGTGTGACTTCTT	114
*EFα1*	XP_002284964.1	GAACTGGGTGCTTGATAGGC	AACCAAAATATCCGGAGTAAAAGA	164

### 2.7 Statistical analysis

Data were analyzed using appropriate statistical tools and are presented as mean ± standard deviation (SD). A two-way ANOVA was conducted to evaluate both main and interaction effects on gene expression levels, and normality and homogeneity of variances were assessed prior to performing the analysis. For the leaf disc assay, data were normalized to the control and a one-sample *t*-test was applied to assess whether treatment values significantly differed from the reference value. *Post hoc* comparisons were performed using Tukey’s Honestly Significant Difference (HSD) test. All analyses were carried out using Statgraphics Centurion 19 (X64 edition; (2022, The Plains, Virginia, United States), with statistical significance set at *p* ≤ 0.05. The statistical analysis of VOCs production was performed using SPSS Win 12.0 (SPSS Inc., Chicago, IL, United States). Pearson correlation coefficients were calculated to assess the relationship between vitality, defined as 0 for growth inhibition within 6 days and 1 for pathogen growth, and VOC production. A critical correlation value of 0.456 was considered (degrees of freedom = 17, α = 0.05). Principal component analysis (PCA) was conducted using Statistica 12 software (StatSoft Inc., Tulsa, OK, United States) on auto-scaled data to explore the variance structure in VOC datasets.

## 3 Results

In this study, a comprehensive approach was developed to evaluate the antagonistic potential of grapevine endophytic populations against *B. cinerea*, combining both *in vitro* and *in vivo* assessments. The investigation followed a stepwise strategy, beginning with a preliminary screening to identify potential BCA candidates through *in vitro* antagonistic assays. These tests assessed their antifungal activity as well as their resistance to commonly used agrochemicals, including copper, commercial fungicides, and sulfur dioxide. In addition, the profile of VOCs was assessed to determine their role in inhibiting pathogen growth.

This characterization phase provided valuable insights into the candidates’ compatibility with various plant protection strategies, supporting their potential use either in fully biological systems or in integrated approaches combining biocontrol and conventional treatments.

Following the initial selection, the most promising strains were further evaluated *in vivo* to confirm their antagonistic activity under more realistic plant-pathogen interaction conditions.

### 3.1 Antagonistic assay

**FIGURE 1:**
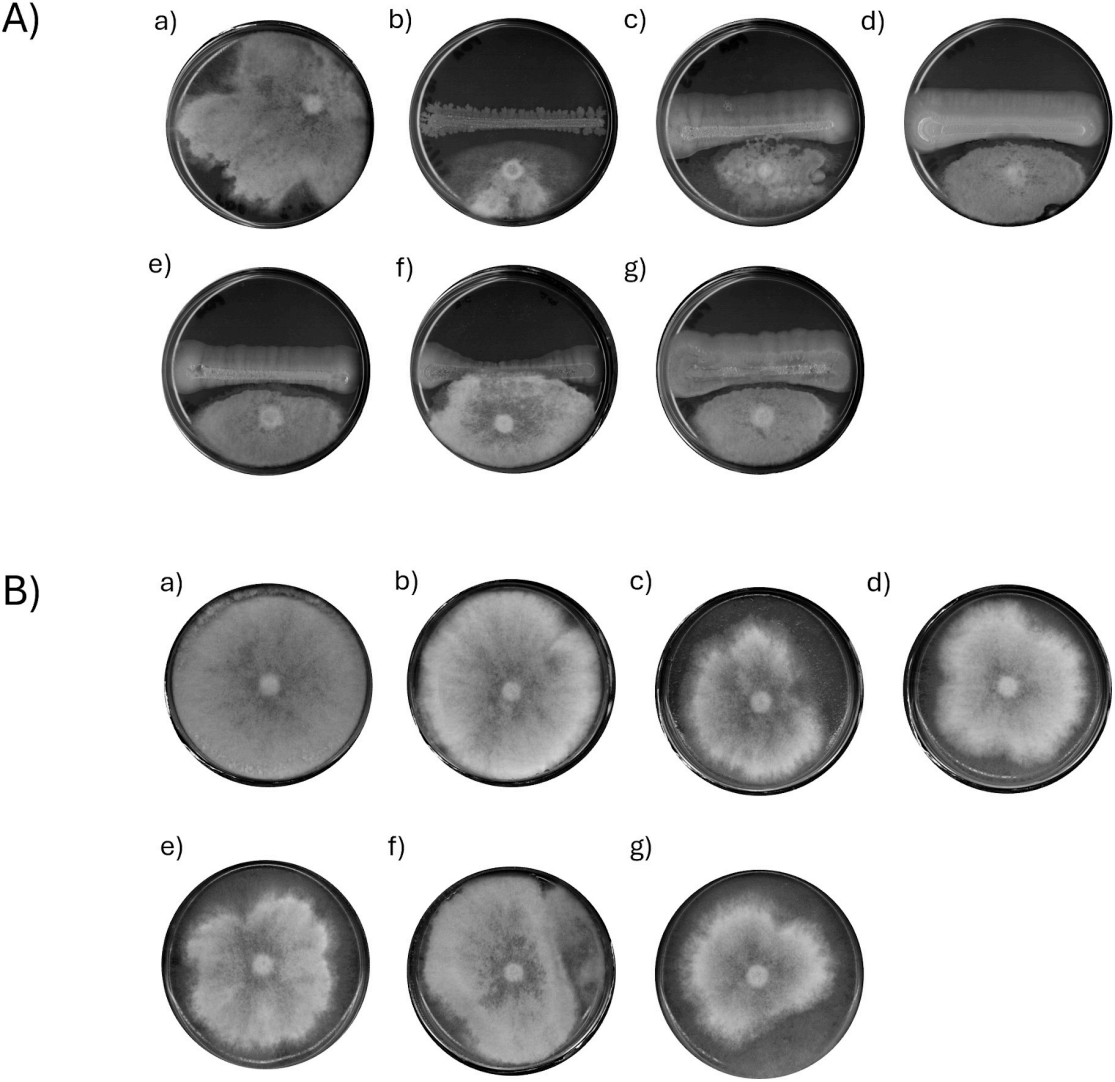
Antagonistic interaction between candidates and *B. cinerea*. **(A)** Dual Culture Plate assay, (a) *B. cinerea* control; *B. cinerea* against (b) ED163; (c) ED203; (d) ED206; (e) ED217; (f) ED219; (g) ED221. **(B)** Double Petri Dish assay, (a) *B. cinerea* control untreated); (b–g) *B. cinerea* co-incubated with (b) ED163; (c) ED203; (d) ED206; (e) ED217; (f) ED219; (g) ED221.

**FIGURE 2:**
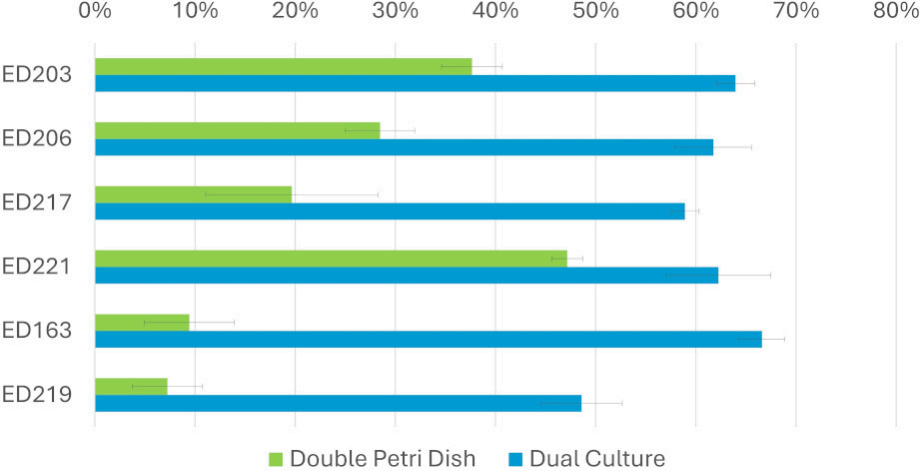
Graph representation of conidial growth inhibition percentage in DC and DPD (%) by the isolated species. Values are expressed as areas mean ± standard deviation of each triplicate determination.

Isolates (Supplementary Table 1) were evaluated *in vitro* to assess their antagonistic activity against *B. cinerea* through two distinct mechanisms: competition for space and nutrients (Dual Culture assay, DC), and production of volatile antifungal compounds (Double Petri Dish assay, DPD). Out of 42 endophytic isolates, 6 demonstrated antagonistic activity by effectively limiting the mycelium growth. The remaining isolates did not exhibit any noticeable impact on mycelium development. Isolates were considered effective in inhibiting *B. cinerea* growth when they showed an inhibition greater than 40% and prevented the overgrowth of their biomass by the pathogen (Figure 1A). Among them, ED163 showed the strongest antagonistic activity, with an inhibition rate of 66.60 ± 2.3%. Isolates ED203 (63.96 ± 1.91%), ED221 (62.26 ± 5.24%) and ED206 (61.75 ± 3.81%) also showed relevant levels of inhibition, all exceeding 60%. ED217 (58.92 ± 1.39%) and ED219 (48.60 ± 4.06%) displayed comparatively weaker inhibition (Figure 2). From this point forward, isolates exhibiting inhibitory activity will be referred to as BCAs.

Since the inhibition of *B. cinerea* observed in the DC assay could result from both direct and indirect cell-to-cell interactions, the isolates that showed inhibitory activity were also tested using the DPD assay, which highlights non-contact, VOC-mediated interactions. This effect was confirmed when mycelium development was significantly reduced compared to the untreated control. All 6 isolates identified in DC assays were tested (Figure 1B). Among them, ED221 and ED203 exhibited the highest inhibitory activity via VOC production, with inhibition rates of 47.16% ± 1.55% and 37.65 ± 3.03%, respectively, suggesting their strong potential as VOC-producing antagonists. The remaining isolates ED206 (28.47 ± 3.03%), ED217 (19.66 ± 8.60%), ED163 (9.43 ± 4.50%) and ED219 (7.24 ± 3.52) showed weaker antagonistic activity. Notably, none of the tested BCAs were able to completely inhibit mycelial growth in the DPD assay (Figure 2).

### 3.2 Tolerance tests

To evaluate the potential practical application of the selected BCA candidates, tolerance assays to copper and the commercial fungicide SWITCH were performed. Regarding the BCAs’ sensitivity to copper, the results showed that none of the candidates were able to grow at a Cu^2+^ concentration higher than 100 mg/L (data not shown). Growth at 100 mg/L Cu^2+^ was observed only for ED203 and ED219, while ED217, ED206, and ED221 were able to proliferate up to 64 mg/L. ED163 was the most sensitive isolate, exhibiting complete growth inhibition even at the lowest tested concentration of copper (Table 2). As far as the fungicide sensitivity, all BCAs were able to tolerate the fungicide up to 1 g/L, except for ED163, which was only able to grow in the absence of the fungicide. Finally, the selected BCAs were tested for their resistance to sulfur dioxide to evaluate their potential survival following field application and possible persistence during the fermentation. In particular, the results indicate that all tested BCAs exhibited significant growth in PDA medium at pH 3.5 (data not shown), but their viability was drastically reduced (<10 CFU/mL) in the presence of sulfur dioxide, suggesting a high sensitivity to this antimicrobial at standard oenological dosage (50 mg/L).

**TABLE 2:** Evaluation of growth capability of BCAs in presence of copper, commercial fungicide Switch and sulfur dioxide.

Identification	ID CODE	Cu^2+^ mg/L				SWITCH			PDA pH 3.5		PDA pH 3.5 + 50 mg/L K_2_S_2_O_5_	
		0	20	64	100	0	0.5 g/L	1 g/L	OD	CFU/mL	OD	CFU/mL
*A. pullulans*	ED203	+	+	+	+	+	+	+	1.27 ± 0.01	≈ 1.27*10^7^	0.09 ± 0.00	< 10
*A. pullulans*	ED206	+	+	+		+	+	+	1.30 ± 0.13	≈ 1.3*10^7^	0.01 ± 0.00	< 10
*A. pullulans*	ED217	+	+	+		+	+	+	0.94 ± 0.06	≈ 9.44*10^6^	0.01 ± 0.00	< 10
*A. pullulans*	ED221	+	+	+		+	+	+	1.07 ± 0.07	≈ 1.07*10^6^	0.02 ± 0.00	< 10
*B. velezensis*	ED163	+				+			0.24 ± 0.02	≈ 1.18*10^7^	0.02 ± 0.00	< 10
*P. extremaustralis*	ED219	+	+	+	+	+	+	+	1.16 ± 0.26	≈ 5,8*10^8^	0.01 ± 0.00	< 10

Tolerance tests were conducted on PDA medium supplemented with increasing concentrations of copper or SWITCH or sulfur dioxide. The symbol (+) was assigned in the presence of visible colony growth.

### 3.3 Candidate selection and *in vivo* antagonistic activity evaluation

#### 3.3.1 Yeast strains identification

**FIGURE 3:**
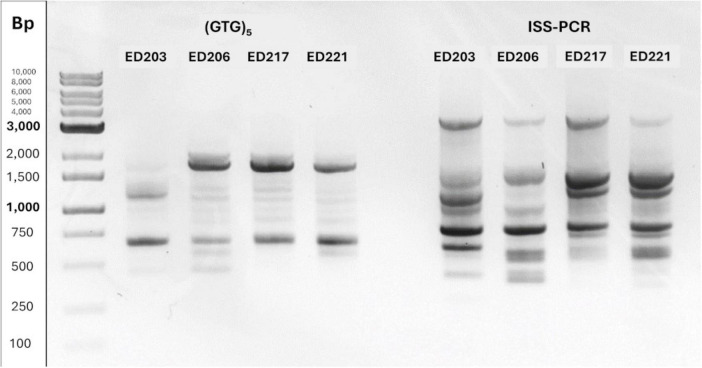
Molecular fingerprinting of *A. pullulans* isolates obtained using (GTG)5-PCR (left) and ISS-PCR (right). The comparison of banding patterns highlighted genetic variability among the isolates.

To discriminate and select the most promising candidate to evaluate the BCAs mode of actions, ISS-PCR and a (GTG)_5_-PCR fingerprinting analyses were applied for *A. pullulans* candidates. As shown in Figure 3, distinct genetic profiles were obtained by amplifying the genomic DNAs of *A. pullulans* isolates using (GTG)_5_ primer (left panel) and the EI1/LA2 primer pair (right panel). The amplification resulted in DNA fragments of varying lengths (in base pairs), and comparison of the banding patterns generated with the two primer sets revealed clear differences among the candidates. Specifically, ED217 and ED221 showed similar band profiles, suggesting their potential ascription to the same strains, whereas ED203 and ED206, exhibiting different profiles, could be considered two different strains. Considering strains profiles and the results obtained from *in vitro* antagonistic activity tests, ED203, ED206, and ED221 strains were selected for subsequent *in vivo* assays.

### 3.4 Volatile organic compounds production

VOC production was assessed to identify the volatile metabolites released by strains and pathogen alone and during BCAs and *B. cinerea* interaction. Specifically, Supplementary Table 2A reports VOCs produced by BCAs and *B. cinerea* individually and Supplementary Table 2B lists VOCs detected during BCA–pathogen co-culture. Supplementary Table 2C reports the difference in VOC production calculated as difference between compounds produced in co-culture and the individual BCA and pathogen contributions; positive values indicate compounds enhanced during interaction, whereas negative values indicate reduced or suppressed production. VOC data were expressed as relative abundances (peak areas), therefore biological interpretations are based on comparative VOC profiles among treatments.

**FIGURE 4:**
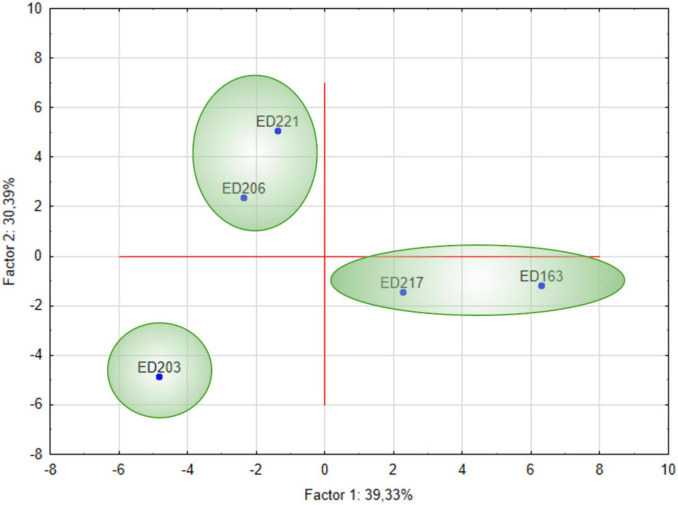
Principal component analysis (PCA) biplot. Illustration of the relationships between BCAs and VOCs. Factor 1 (x-axis) and Factor 2 (y-axis) explain 39.33 and 30.39% of the total variance, respectively. The distribution of BCAs and VOCs along these two factors highlights potential correlations between specific strains and their metabolic profiles.

**FIGURE 5:**
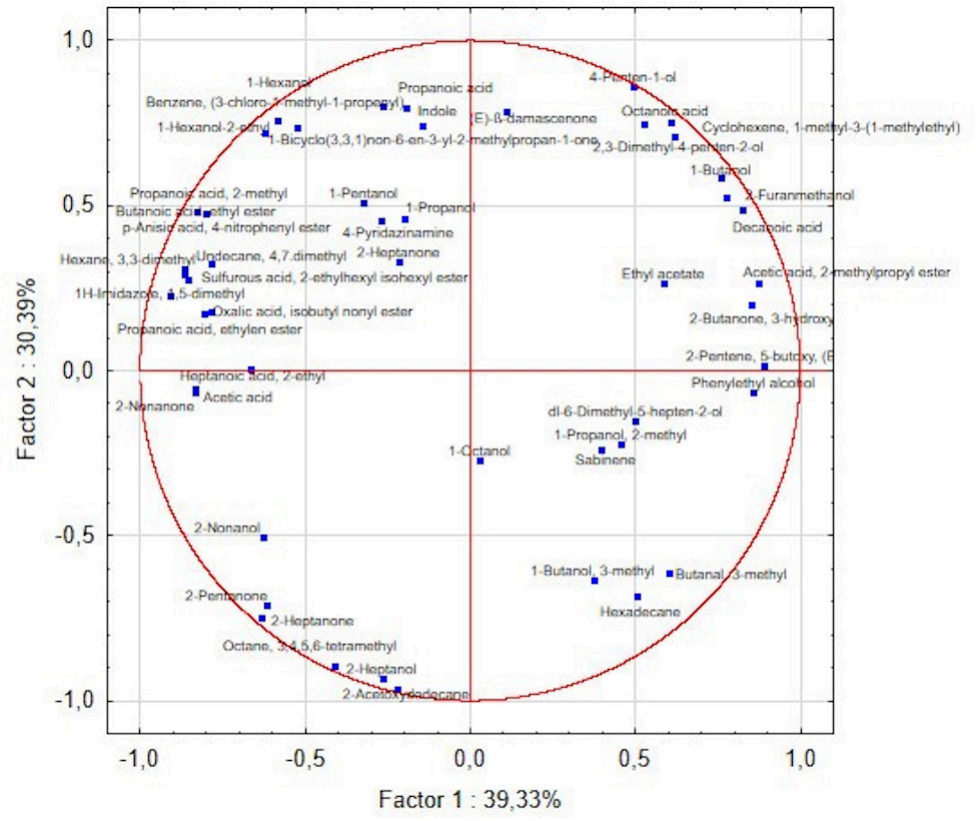
Principal component analysis (PCA) with BCA clusters. Scores plot displaying the distribution of the five BCA strains based on their VOC production patterns. Separation among clusters indicates distinct VOC profiles associated with different BCAs.

The PCA revealed that two factors explained 69.72% of the total variance (Figure 4). Based on BCAs VOC profiles, three distinct clusters of microorganisms were identified (Figure 5), reflecting differences in their metabolic responses. The first cluster included ED206 and ED221, which was characterized by the production of alcohols and aromatic compounds such as 1-hexanol, 1-hexanol-2-ethyl, 4-penten-1-ol, indole and β-damascenone, octanoic acid, and cyclohexene derivatives. The second cluster comprised ED217 and ED163 strains were characterized by the production of alcohols, esters and terpenes such as ethyl acetate, acetic acid, 2-methylpropyl ester, 2-butanone (3-hydroxy), phenylethyl alcohol, sabinene and 2-pentene derivatives. The third cluster consisted of strain ED203, which exhibited a VOC profile mainly composed of ketones, alcohols and hydrocarbons, such as 2-pentanone, 2-heptanone, 2-heptanol, 2-nonanone, and octane.

While PCA was used to discriminate BCA strains based on their overall VOC emission profiles, VOCs associated with *B. cinerea* alone and with BCA–pathogen interaction were interpreted through a comparative analysis of compound presence and modulation, as the biological focus was on interaction-specific changes rather than strain clustering. A total of 70 VOCs were identified, including 8 acids, 15 alcohols, 4 aldehydes, 7 alkanes, 7 alkenes, 12 esters, and 9 ketones. Some VOCs were detected exclusively in the *B. cinerea* treatment, others only in the presence of the BCAs, and several were produced specifically during the BCA–pathogen interaction. In the first case, different compounds were generated by *B. cinerea* in control treatment, such as hexadecane, octanoic acid, udecane, 3,8-dimethyl, acetic acid, 1-hexanol-2-ethyl and benzaldehyde, but not all these components were produced even during the interaction between the pathogen and the different BCA candidates.

Notably, some VOCs were predominantly detected during the interaction between *B. cinerea* and potential BCAs as certain alcohols (2-heptanol, 1-hexanol, 2-nonanol, 2-furanmethanol), ketones (2-heptanone, 2-nonanone) and alkenes [2-Pentene, 5-butoxy, (E)].

### 3.5 *In vivo* tests on grape leaves

**FIGURE 6:**
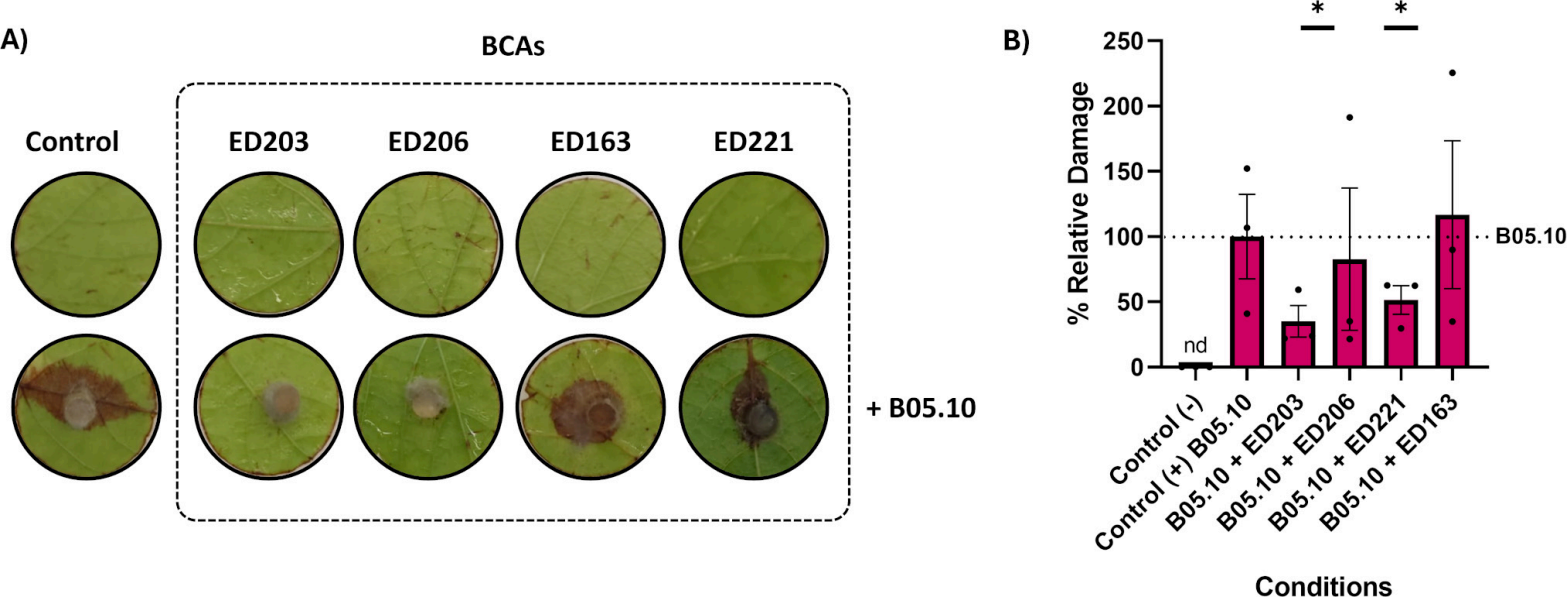
*In vivo* results of BCAs against *B. cinerea* on grapevine leaves. **(A)** Representative images of grape leaf discs post inoculation with *B. cinerea* alone (control) or co-inoculated with different BCA strains. **(B)** Quantification of necrotic damage on leaf discs expressed as surface percentage of necrosis. Each bar represents the mean ± SD of three biological replicates (*n* = 3), each corresponding to one leaf disc obtained from a different leaf. * indicates a statistically significant difference in damage compared with the positive control (*B. cinerea* only) (*p* < 0.05).

The selected BCA candidates were evaluated for their capability to limit the *B. cinerea* strain B05.10 infection *in vivo* on grape leaves. For the *in vivo* trials on grape leaves, pathogen growth was assessed by examining the necrosis status of the leaf tissue (Figure 6A). The results showed considerable variability between replicates, which limits the strength of the conclusions. Nevertheless, a general trend toward reduced damage was observed in discs treated with BCAs compared to the positive control (*B. cinerea* only). In particular, the strains *A. pullulans* ED203 and *A. pullulans* ED221 led to a statistically significant reduction in damage (*p* < 0.05), highlighting their potential as effective biocontrol agents (Figure 6B).

### 3.6 Interaction BCA/host/pathogen

#### 3.6.1 Callus formation and interaction observation by electron microscope

**FIGURE 7:**
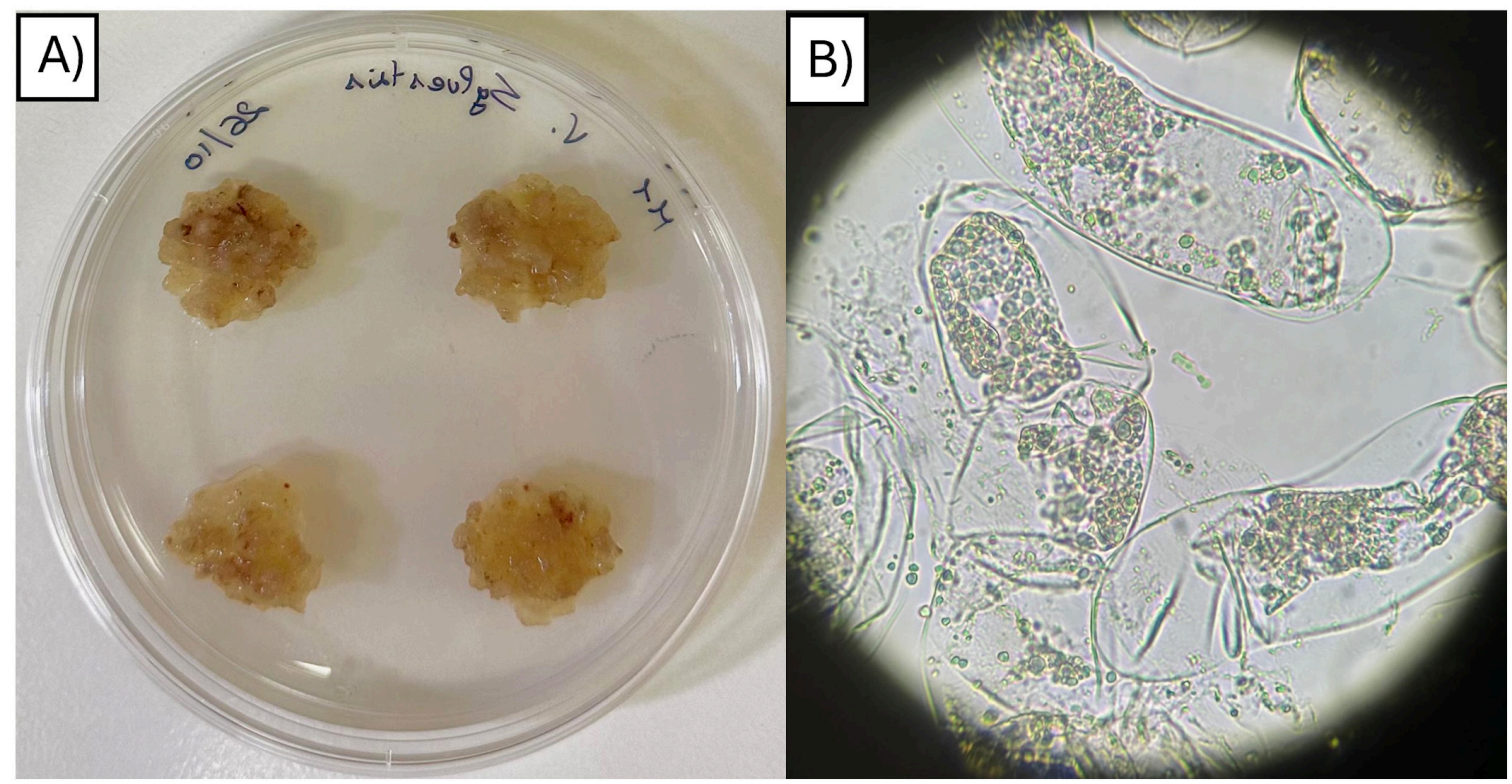
*V. sylvestris* leaves cells. **(A)** Five months *V. sylvestris* callus, **(B)** *V. sylvestris* liquid-culture cells observation under optical microscope.

Callus tissue was successfully induced from surface-sterilized *V. sylvestris* leaves. The success for sterilizing treatment was determined as callus growth without contamination. Initially, the leaf explants produced yellowish calli with smooth surfaces and irregularly shaped cells, as evidenced by microscopic observation. After 1 month of culture, the callus surface became more granular and spherical in texture, with a more intense yellow coloration (Figure 7). Browning of the callus was indicative of tissue death or the formation of fibrous callus, which is characterized by a denser and more compact structure.

**FIGURE 8:**
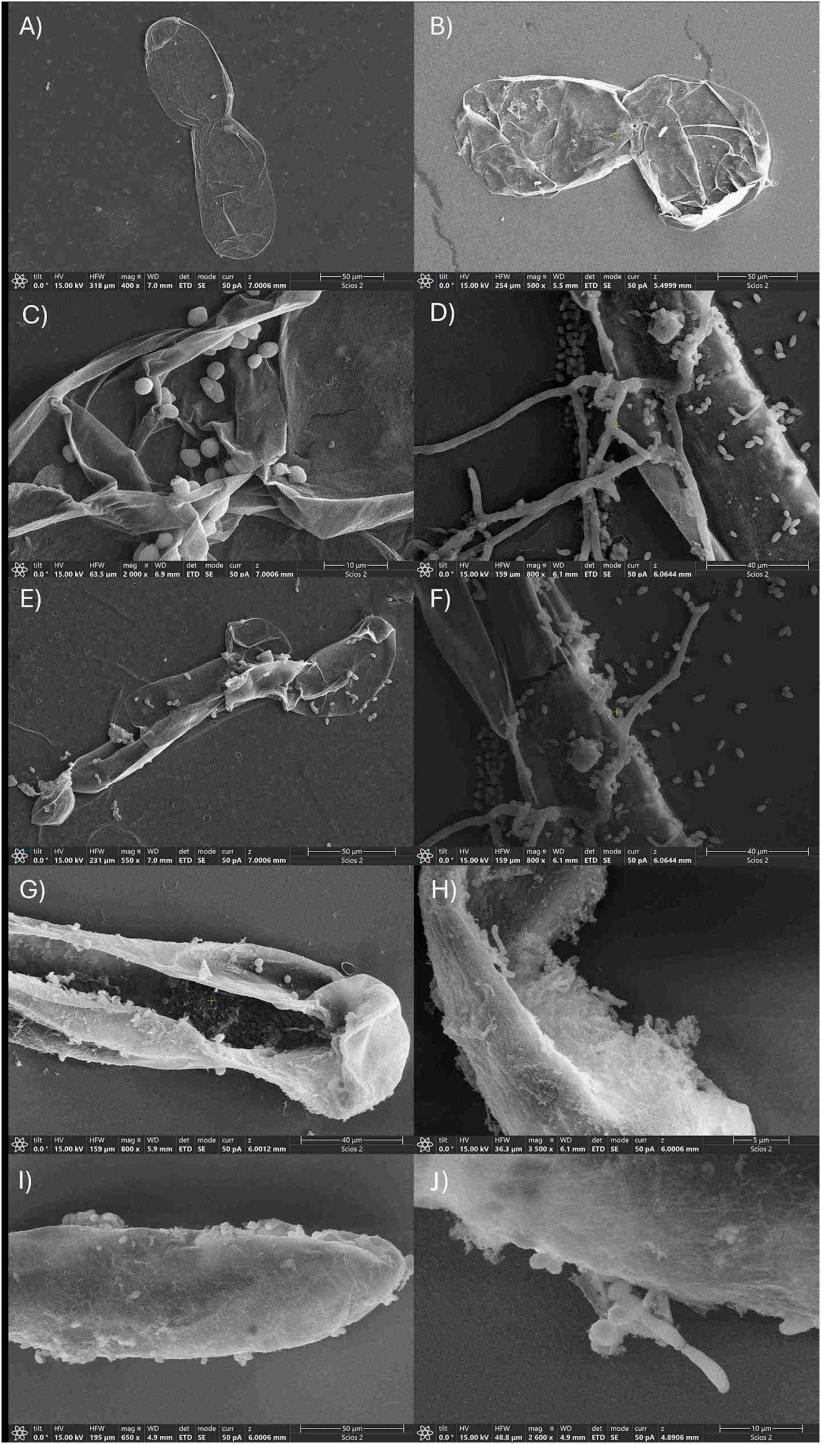
Scanning electron micrographs of suspension cultures of *V. sylvestris* cells, *B. cinerea* spores and BCA 4 and 8 days after elicitation. **(A)** *Vitis* cells without elicitation, day 4 **(B)** *Vitis* cells without elicitation, day 8 **(C)** *Vitis* cells elicited with *B. cinerea* B05.10, day 4 **(D)** *Vitis* cells elicited with *B. cinerea* B05.10, day 8 **(E)** *Vitis* cells elicited with *B. cinerea* UCA992, day 4 **(F)** *Vitis* cells elicited with *B. cinerea* UCA992, day 8 **(G)** *Vitis* cells elicited with B05.10 and co-inoculated with ED163, day 4 **(H)** *Vitis* cells elicited with B05.10 and co-inoculated with ED163, day 8 **(I)** *Vitis* cells elicited with UCA992 and co- inoculated with ED163, day 4 **(J)** *Vitis* cells elicited with UCA992 and co-inoculated with ED163, day 8.

Liquid culture allowed for easier visualization of callus tissues in contact with the pathogen and BCAs under the SEM. Observations at 400/500 × magnification revealed that grapevine cells not subjected to elicitation were generally elliptical and formed aggregates while the wall surface appeared flat, with a fibrillar, mesh-like structure. No evident differences in cell morphology or wall structure were observed between samples collected at 4 and 8 days of cultivation (Figures 8A,B). Observing cultures 4 and 8 days after elicitation with *B. cinerea*, spores of both pathogen strains were in contact with grapevine cell surfaces, either individually or in small clusters, with evident germination and hyphal elongation in the control samples (Figures 8C,E). Otherwise, a progressive increase in mycelial development was observed over time, with hyphae appearing longer, more branched, and densely distributed, indicating an active colonization of the plant tissue (Figures 8D,F). In contrast with the culture with only *B. cinerea*, in the co-culture with *B. velezensis*, fungal growth remained notably limited. While *B. cinerea* spores were still detectable on the plant surface, the formation and extension of hyphae were significantly reduced. Instead, bacterial cells produced a kind of patina able to cover the grape surface (Figures 8G–J). The distribution of cells on host was variable and lacked a defined spatial pattern, with most cells forming clusters or microcolonies.

#### 3.6.2 *STS1* and *VvABCG44* gene expression

The gene expression of *STS1*, involved in stilbene biosynthesis, and *VvABCG44*, a transporter gene potentially involved in the mobilization of phenylpropanoid compounds (Pietrowska-Borek et al., 2021), were assessed in plant cells collected at 4- and 8-days post-elicitation. To confirm their presence in *V. sylvestris* genomic DNA, the potential *loci* (Table 1) were amplified and sequenced; the results confirmed that the target genes have an identity percentage > 87% when compared to the corresponding *V. vinifera* sequences. This evidence supported the use of *V. vinifera*-based gene models as valid references for gene expression analysis.

**FIGURE 9:**
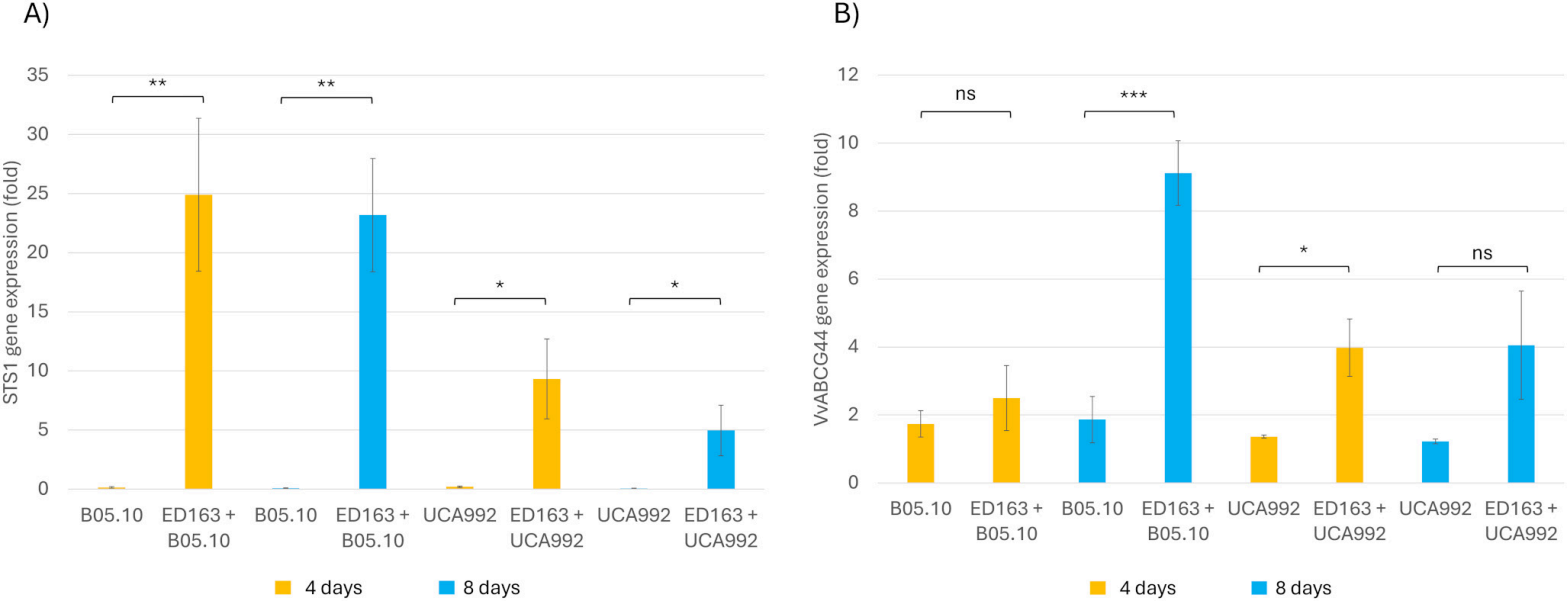
Expression of phenylpropanoid pathway genes. Cells of *V. sylvestris* were inoculated with ED163 and two different strains of *B. cinerea* (B05.10 and UCA 992). EFα1 was used as an endogenous control. The expression level of genes in the control cells was set to 1. Values represent the mean ± standard deviation of the three biological replicates. A one-tailed ANOVA revealed statistically significant variations, as indicated by the different levels of significance (*, **, ***). **(A)** Stilbene synthase gene (*STS1*). **(B)** Resveratrol transporter gene (*VvABCG44*).

A statistically significant upregulation of *STS1* was observed in grapevine cells co-inoculated with *B. cinerea* and *B. velezensis* ED163 compared to cells inoculated with *B. cinerea* alone (Figure 9A). After 4 days, *STS1* expression increased approximately 25-fold in the ED163 co-culture relative to the B05.10 control. This induction further increased to approximately 23-fold at 8 days. A similar statistically significant trend, though less pronounced, was observed in the presence of *B. cinerea* strain UCA992, with a 9-fold increase on day 4 and a slight reduction to 5-fold by day 8.

Accumulation of trans-resveratrol induces gene expression of the ABCG transporter, which is associated with the transport of this stilbene compound in plants, for this reason *VvABCG44* gene expression was evaluated (Pietrowska-Borek et al., 2021). Specifically, plant cell cultures inoculated with *B. cinerea* B05.10 and ED163 did not show a statistically significant increase of the gene expression after 4 days compared with suspension contained only pathogen, while a statistically significant increase of around 9-fold was observed at 8 days post inoculum in the plant cell cultures inoculated with *B. cinerea* B05.10 and ED163 compared with suspension contained only pathogen. A statistically significant increase was observed even in co-culture with ED163 and *B. cinerea* UCA992 at 4 days (4-fold) compared with the control. However, this increase remained stable from day 4 to day 8, with no statistically significant differences in this time-frame (Figure 9B).

A two-way ANOVA revealed several key findings regarding the factors influencing gene activation in the co-culture system (Supplementary Table 3 and Supplementary Figure 2). Firstly, the presence of the BCA in co-culture compared with the control had a highly significant effect on gene activation (*p <* 0.001).

Analyzing the two target genes, a significant difference in their activation was detected (*p =* 0.0149), suggesting gene-specific responses. Lastly, no significant differences in gene activation were observed across the post-inoculation time points considered (*p* > 0.05).

## 4 Discussion

In a sustainable and innovative approach to vineyard management, BCAs represent a promising strategy for controlling pests and diseases. However, the characterization of candidate strains is essential to optimize their effectiveness under field conditions. Indeed, due to low pH, excess sugar and polyphenol content, grapes are a challenging environment for microbial proliferation (Rantsiou et al., 2020). Therefore, *in vivo* applications may further restrict BCA growth.

Although scientific literature is progressively expanding with studies elucidating the mechanisms of action of selected BCAs and providing *in field* evidence of their efficacy against grapevine pathogens (Galli et al., 2024; Palmieri et al., 2022), further research is required to elucidate the molecular mechanisms underpinning the tripartite interaction among the plant, the pathogen, and a selected biocontrol agent. The general aim of this work is to advance knowledge within this intermediate ground by investigating microbe-plant interactions in the context of their interplay with the pathogen. This objective was pursued using SEM observation and *in vitro* grapevine cell cultures, which allow for the standardization and reproducibility of experimental results. To achieve this overarching goal, the study pursued specific objectives: (i) to identify the most promising antagonistic strains for potential field applications, and (ii) to assess whether integration with conventional treatments, such as fungicides or copper-based products, affects BCA viability and activity. Moreover, adopting a holistic approach, an additional objective was to determine, from a winemaking perspective, the sensitivity of BCAs to sulfur dioxide (SO2), the principal antimicrobial compound used during wine production.

The evaluation of antagonistic activity against *B. cinerea* strain B05.10 in laboratory conditions is a crucial step in identifying potential effective BCAs. In the present study, among the 25 species analyzed, only *A. pullulans* and *B. velezensis* exhibited significant inhibitory activity against *B. cinerea*.

The role of *A. pullulans* as a BCA against *B. cinerea* has been widely documented, with several studies highlighting its effectiveness (Agarbati et al., 2022; Sepúlveda et al., 2023). In addition, antagonistic activity against various *Penicillium* species, such as *P. expansum, P. digitatum, P. glabrum* (Agirman and Erten, 2020; Cabañas et al., 2020), as well as against *Aspergillus* species, including *A. tubingensis* and *A. carbonarius* (Dimakopoulou et al., 2008; Pantelides et al., 2015) has also been shown. This yeast species produces complex carbohydrates (e.g., pullulan, β-glucans), hydrolytic enzymes, cyclic depsipeptides and siderophores that inhibit enzymes such as xylanase, an enzyme directly related to the virulence of necrotrophic pathogens such as *B. cinerea* (Di Francesco et al., 2021). Consistently with PCA clustering, *A. pullulans* strains were characterized by VOC profiles enriched in alcohols and esters, compound classes widely reported for their antifungal activity and their role in microbial antagonism against phytopathogens. Compounds detected as 1-butanol-3-methyl and 1-propanol-2-methyl, are known to be emitted in response to pathogens such as *B. cinerea*, *Colletotrichum acutatum*, and *Penicillium* spp. (Di Francesco et al., 2015). As discussed by Ali et al., VOC production is a strain-dependent trait that can be strongly influenced by environmental factors such as microenvironmental conditions, pH, nutrient availability, and interactions within the microbial community (Ali et al., 2025). This concept is supported by the comparison between ED217 and ED221, which, despite displaying highly similar banding profiles indicative of close genetic relatedness, exhibited distinct VOC emission patterns. Furthermore, the two isolates were obtained from different *V. sylvestris* plants sampled at distinct locations (Pizzi et al., 2025), suggesting that host-associated and micro-environmental factors may have contributed to the observed functional divergence. Notably, *A. pullulans* ED221 produced monoterpenes such as sabinene, which is known for its antifungal properties and have been reported as metabolite produced by endophytic fungi (Walther et al., 2021). Generally, terpenes have been shown to inhibit pathogens like *Alternaria solani*, according to Ivănescu et al. (2021). Ricciardi et al. observed the presence and biocontrol activity of valencene in grapevine leaves, identifying the *VvVal* gene as crucial for valencene synthesis. When tested *in vivo*, this compound could reduce *Plasmopara viticola* sporulation by 53–100%. The occurrence of valencene in grape tissue is likely due to its endophytic community (Ricciardi et al., 2021). Since sabinene is also a plant-derived secondary metabolite (Judžentienė et al., 2024), its detection may reflect either direct microbial synthesis or a plant metabolic response potentially stimulated by the presence of the endophyte. While specific microbial synthesis pathways for 1,5-dimethyl-1H-imidazole are not well documented in the present results, its chemical synthesis and the microbial production of structurally related imidazole derivatives have been described in various biological contexts (Rani et al., 2022). Further investigations will therefore be required to determine whether a dedicated microbial pathway for this compound exists. Similarly, compounds such as 1-bicyclo (3,3,1)non-6-en-3-yl-2-methylpropan-1-one, typically associated with synthetic or fragrance chemistry (Roy et al., 2023) display uncertain or unusual biological origins, reflecting probably poorly characterized metabolic reactions, analytical artifacts or background contaminants. Conversely, sulfurous acid, 2-ethylhexyl isohexyl ester has already been reported in plant matrices (Shaheen Khan et al., 2019; Wiraswati et al., 2023), making its biological detection plausible, although its specific biosynthetic pathway remains poorly understood.

Observing the compounds produced by the pathogen and BCAs alone, this study even shows how several VOCs were detected only in the *B. cinerea* monoculture and were not produced during the BCA–pathogen interaction. This result suggests that the presence of the BCAs affected the pathogen’s metabolism, reducing or preventing the release of some compounds that normally are emitted when growing alone. Notably, the BCA–pathogen interaction was associated with a shift in the overall VOC profile, characterized by compounds such as 1-hexanol, 2-heptanol, 2-nonanol, 2-heptanone and 2-nonanone. This pattern differs from the more complex and strain-specific VOC profiles observed in BCA monocultures, suggesting that microbial interaction modulates volatile emission rather than reflecting constitutive BCA metabolism. Overall, VOCs production should be interpreted with caution, and targeted analytical approaches would be needed to confirm their biological relevance.

The presented data even supports the promising use of *A. pullulans* as a BCA, considering that all sensitivity tests indicate a potentially positive role in the viticulture framework. In summary, the selected *A. pullulans* strains were resistant to viticultural dosages of the fungicides SWITCH (Sygenta) and copper, while exhibiting sensitivity to oenological levels of SO2. Sensitivity to commercial fungicides was investigated by Wang et al. studying indigenous yeast in vineyards, and *A. pullulans* showed, with other species as *M. pulcherrima* and *S. cerevisiae*, the ability to growth on fungicide Vivando (AP—metrafenone) up to 1 mg/mL but was inhibited by Pristine (pyraclostrobin and boscalid) and Procure (triflumizole) (Wang et al., 2018). Moreover, the effect of organic fungicides in vineyards was evaluated by Comitini and Ciani (2008) and *A. pullulans* was the predominant yeast species on grapes, followed by *Criptococcus* spp., and its presence was confirmed in two consecutive vintages. Inorganic fungicide treatment as sulfur and copper sulfate was evaluated in the same study, showing also in this case a significant presence of *A. pullulans*. Several studies suggest *A. pullulans* dominance in copper tolerance tests (Grangeteau et al., 2017; Martins et al., 2018; Rantsiou et al., 2020) and can be explained by the melanin production by this species against the biosorption of copper (Gadd and de Rome, 1988).

*A. pullulans* is consistently prevalent across various vineyards, regardless of the grape varieties cultivated and the fungicide regimens employed. This suggested that the diverse reactions of *A. pullulans* as BCA could be attributed to the specific compounds present in the treatments (Rantsiou et al., 2020). In conclusion, application of organic and inorganic fungicides in vineyards could result in a dramatic reduction of yeast population and a shifting toward *A. pullulans* dominance, able to survive a different variety of commercial fungicide in different concentrations. As confirmed by this study, presence can be controlled by application of SO_2_, being sensitive to a standard oenological dosage. Otherwise, the results confirmed that *A. pullulans* also present *in vivo* inhibition potential against *B. cinerea*, forming only a small circumference of necrosis close to the mold plug, supporting what Ayogu et al. (2023) have reported during *in vivo* assays on grapevine leaves against gray mold, thus demonstrating its effective antifungal activity even on plant substrates.

More information is available about bacteria as biocontrol agents, with most commercially available biological products for vineyard applications formulated using *Bacillus* sp. as the active ingredient (Junaid et al., 2013). Commonly employed species include *B. subtilis, B. amyloliquefaciens, B. licheniformis*, and *B. pumilus* (Veras et al., 2023). Several studies have showed the potential of *B. velezensis* as biocontrol agent on grape and other crops (Cui et al., 2020; Li et al., 2022) against pathogens as *B. cinerea* (Toral et al., 2020), *A. flavus* (Wang et al., 2023) *A. carbonarius* (Silveira et al., 2024) and *P. digitatum* (Vu et al., 2023). Volatile organic compounds evaluation against *B. cinerea* revealed the production of compounds with antifungal properties already detected in other studies. As shown in this study, *Bacillus* strains predominantly release alcohols already known for their antimicrobial or antifungal properties (Li et al., 2015), such as 2- butanone, 3-hydroxy, whose production was previously reported by Silveira et al. during assays against *A. carbonarius* (Silveira et al., 2024), and 2-phenylethanol, produced by *Serratia liquefaciens* with an antagonistic effect against *B. cinerea* (Wei et al., 2025). Notably, compounds such as 2-furanmethanol and 2-pentene, 5-butoxy, (E) were exclusively detected in co-culture, as these compounds were not observed in the *B. cinerea* control nor in BCA monocultures, indicating that microbial interaction can lead to the emergence of novel volatile compounds. However, the application of *B. velezensis* in integrated pest management programs may be limited, as the species exhibits high sensitivity to copper and commercial fungicides at relatively low concentrations (20 and 0.5 mg/L, respectively). These findings are consistent with those reported by Vörös et al. (2019), who observed that *B. velezensis* is sensitive to heavy metals such as copper, nickel, zinc, and cadmium. Additionally, their study showed that sulfonylurea herbicides severely inhibited bacterial growth, even at concentrations as low as 6.25 mg/L. Nevertheless, the application of SO_2_ at standard oenological dosages appears sufficient to prevent the proliferation of *B. velezensis* during subsequent winemaking processes. Finally, the efficacy of the selected candidates in mitigating *B. cinerea* infection was evaluated *in vivo* trials, revealing significant differences in the BCAs’ performance. Biological control using plant-associated microorganisms represents a promising and environmentally sustainable strategy for disease management (Jiang et al., 2018). Several studies have reported positive results using *B. velezensis* as a biocontrol agent *in vivo* tests on grapevine leaves. For instance, Hamaoka et al. (2021) demonstrated that *B. velezensis* effectively reduced symptoms of gray mold caused by *B. cinerea*, anthracnose by *C. gloeosporioides*, and downy mildew by *P. viticola* on grape disk leaves throughout efficient antagonistic activity.

To investigate the interaction among a potential BCA, pathogen and host, grapevine cell cultures were established using *B. velezensis* as the best candidate identified for its antagonistic properties in this work. The *in vitro* approach allows for the rapid and reproducible production of biological material, enabling detailed investigation of plant-pathogen-microbe interactions under controlled conditions. Compared to whole-plant assays, cell cultures provide a faster and more manageable system for studying responses to pathogens and environmental stresses. It is important to underline that callus-based cell culture represents a simplified experimental system and does not fully describe the complexity of whole-plant or field-level defense responses, which are influenced by tissue differentiation, systemic signaling and environmental influences (Jiang et al., 2018).

Following the attempts to induce callus formation from both grapevine leaves species, successful calli were obtained from leaf explants of *V. sylvestris*. *V. vinifera*, and *V. sylvestris* belong to the same genus and share a significant portion of their genome. *V. sylvestris* is considered the wild ancestor of the cultivated *V. vinifera* and the morphological differences are primarily attributed to the domestication process (De Andrés et al., 2012; Zdunić et al., 2020). The choice to consider the *V. vinifera* genome as a reference was guided by its high level of annotation and the broad availability of supporting literature. Among the *Vitis* species, *V. vinifera* is the most extensively studied, offering a well-established genomic framework for selecting target genes. In contrast, genomic information for *V. sylvestris* remains limited, making *V. vinifera* a practical starting point for experimental design. Therefore, to ensure the validity of this approach, the identity of genes was confirmed, revealing the *V. sylvestris*-derived calli a suitable reference model for subsequent experiments aimed at investigating the interaction between biocontrol agents, pathogens, and the host plant.

Interaction mechanisms were investigated by electron microscope observation and gene expression analysis. Microscope observations revealed damaged and dehydrated regions in the callus structure, which can be attributed to artifacts introduced by the sample preparation process, including chemical fixation, dehydration, and critical point drying (McCann et al., 1990). The interaction between microorganisms and various plant cell types to assess their effects on plant health and to evaluate their potential for large-scale plant propagation has been shown. Souissi et al. (1997) used scanning electron microscopy to examine uninoculated callus tissues from leafy spurge (*Euphorbia esula* L.), which exhibited elongated plant cells with smooth surfaces, small shallow pits and variably shaped particulates. After 48 h of inoculation, many rod-shaped, bacterial biofilm-like structures were found attached to the cell surfaces of the inoculated callus tissue. However, the distribution of bacteria on the surface was uneven, with some regions containing more bacteria than others. This observation is confirmed by the present study where bacteria are scattered on the grapevine cell surface. Nevertheless, contrary to what described (Souissi et al., 1997), bacteria cells are mainly organized in clusters suggesting a specific mechanism of cell-to-cell interaction (i.e., biofilm, lectin-like aggregation, etc.). Souissi et al. (2018) investigated the impact of co-cultivating *Fouquieria splendens* callus with an endophytic bacterium and found no visible damage, discoloration, or growth inhibition after 19 and 45 days compared to non-inoculated controls. On the contrary, the presence of the endophyte enhanced callus biomass. SEM also revealed morphological changes, including the presence of an extracellular matrix, which may reflect a complex interplay between plant growth regulators and bacterial phytohormones during co-cultivation.

In addition to the evaluation of the physical interaction between host and BCA, *B. velezensis* was assessed for its ability to stimulate plant defense responses, specifically through the activation of genes in the phenylpropanoid pathway, which leads to the production of molecules such as resveratrol, that protect plants against a wide range of biotic and abiotic stresses. This study demonstrates that inoculation of *V. sylvestris* cell suspensions with *B. velezensis*, in the presence of *B. cinerea*, induces a significant upregulation of genes involved in the biosynthesis of stilbenes. Previous studies have documented the elicitation of the phenylpropanoid pathway in grapevine cell cultures using compounds such as cyclodextrins and methyljasmonate (Lijavetzky et al., 2008). However, to the best of our knowledge, this is the first work about elicitation with alive cells of pathogen and biocontrol agents in contact with *V. sylvestris* cell culture. Among the genes analyzed, *STS1* exhibited the most pronounced increase in expression, showing a significant upregulation compared to cells inoculated with *B. cinerea* spores. These results strongly suggest that the BCA plays a crucial role in modulating the plant’s immune response by enhancing the expression of defense-related genes. Moreover, the differential expression patterns observed among the analyzed genes reflect the involvement of distinct regulatory mechanisms and signaling pathways, underscoring the complexity of the plant’s defense network. Pietrowska-Borek et al. (2021) reported that cyclodestrins act as elicitors in *V. vinifera* cells, promoting the accumulation of trans-resveratrol and trans-piceid in both cells and culture medium. Interestingly, while they observed a decrease in *VvABCG44* expression after 72 h, the present study shows a sustained expression of the same gene beyond the initial treatment phase. This prolonged expression suggests an extended defensive response to the elicitation, which is consistent with the observed accumulation of trans-resveratrol, a well-known phytoalexin indicative of the plant’s activation of stress-response pathways. Overall, the laboratory- and greenhouse-based assays conducted in this study represent controlled systems that provide a foundation for analyzing specific plant–microbe interactions. However, they also entail limitations, as their translation to vineyard conditions remains challenging due to factors such as environmental variability, the presence of an intrinsic plant-associated microbial community, and constraints related to formulation and delivery. Therefore, scale-up studies will be necessary to confirm the activity of biocontrol agents and to pave the way for their use as complementary tools within integrated disease management strategies with reduced chemical inputs.

## 5 Conclusion

In conclusion, this study offers significant evidence for the potential future use of biocontrol agents to reduce reliance on synthetic fungicides and provides novel insights into the still-fragmented understanding of microbial interactions within the grapevine microbiota. Species like *B. velezensis* and *A. pullulans* were confirmed as endophytes with an effective biocontrol activity against different pathogens, but their application in real condition is still an open question. BCA selection for field applications must align with the specific vineyard management strategy to ensure efficacy and compatibility in the agricultural context. Furthermore, this study highlights the role of VOC-mediated interactions as an integral component of the biocontrol strategy, demonstrating that strain-specific VOC profiles contribute to pathogen suppression while also reflecting functional traits potentially linked to host interaction. Although *B. velezensis* emerged as the most promising BCA based on *in vitro* results, its chemical sensitivity limits its suitability for an integrated pest management strategy. Nevertheless, cell co-culture has enabled to highlight an effective interaction between grape leaves cells, *B. cinerea* and *B. velezensis* as candidate models. The electron microscopy observation revealed formation of a biofilm-like coating by the *B. velezensis* on the callus surface limiting the attachment of pathogen spores, showing a potential mechanism by which this candidate may exert its antagonistic effects against the pathogen. This hypothesis was confirmed by gene expression analysis demonstrating that BCA presence significantly upregulated the expression genes involved in the synthesis and transport of stilbene compounds, suggesting that the biocontrol agent not only inhibits pathogen growth directly but also stimulates the plant’s innate immune responses, enhancing its ability to counteract infections. Importantly, this study provides the first evidence that plant defense responses can be effectively elicited using a biologically realistic system involving viable pathogen spores in combination with living biocontrol agents. Unlike conventional approaches based on chemical elicitors, this strategy demonstrates that BCAs can enhance host defense activation, as shown by the upregulation of phenylpropanoid-related genes, thereby contributing to disease control not only through direct pathogen inhibition but also through stimulation of the plant’s innate immune responses. Finally, this study highlights the challenges of translating from *in vitro* to *in vivo* applications, but it serves as a foundational step for future research and field trials aimed at refining and applying these biocontrol strategies under real agricultural conditions.

## Data Availability

The datasets presented in this study can be found in online repositories. The names of the repository/repositories and accession number(s) can be found in the article/Supplementary material.
