# Correction: Study of grapevine endophytes' interaction with the host and their potential as biocontrol agents as sustainable alternative to agrochemicals

**DOI:** 10.3389/fmicb.2026.1815918

**Published:** 2026-03-09

**Authors:** Simona Pizzi, Alessandra Di Canito, Elena Palencia Mulero, Carmen Cris De Oliveira Nobre Bezerra, Valentina Ricciardi, Giuliana Maddalena, Evelyn Maluleke, Roberto Carmine Foschino, Silvia Laura Toffolatti, Daniela Fracassetti, Gabriella De Lorenzis, Gustavo Cordero-Bueso, Mathabatha Evodia Setati, Ileana Vigentini

**Affiliations:** 1 Department of Biomedical, Surgical and Dental Sciences (DiSBIOC), Università degli Studi di Milano, Milan, Italy; 2 Department of Agricultural and Environmental Sciences (DiSAA), Università degli Studi di Milano, Milan, Italy; 3 South African Grape and Wine Research Institute, Stellenbosch University, Stellenbosch, South Africa; 4 Department of Food, Environmental and Nutritional Sciences (DeFENS), Università degli Studi di Milano, Milan, Italy; 5 Department of Biomedicine, Biotechnology and Public Health, Faculty of Sciences, University of Cádiz, Puerto Real, Spain

**Keywords:** *Aureobasidium pullulans*, *Bacillus velezensis*, *Botrytis cinerea*, elicitation, host interaction, sustainability

In the published article, there is a mistake in the affiliation assignment of Simona Pizzi.

The affiliations were displayed as:

Simona Pizzi^1, 2^, Alessandra Di Canito^1^, Elena Palencia Mulero^1^, Carmen Cris De Oliveira Nobre Bezerra^1^, Valentina Ricciardi^3†^, Giuliana Maddalena^3^, Evelyn Maluleke^4^, Roberto Carmine Foschino^1^, Silvia Laura Toffolatti^3^, Daniela Fracassetti^2^, Gabriella De Lorenzis^3^, Gustavo Cordero-Bueso^5^, Mathabatha Evodia Setati^4^ and Ileana Vigentini^1*^

1Department of Biomedical, Surgical and Dental Sciences (DiSBIOC), Università degli Studi di Milano, Milan, Italy,

2Department of Food, Environmental and Nutritional Sciences (DeFENS), Università degli Studi di Milano, Milan, Italy,

3Department of Agricultural and Environmental Sciences (DiSAA), Università degli Studi di Milano, Milan, Italy,

4South African Grape and Wine Research Institute, Stellenbosch University, Stellenbosch, South Africa,

5Department of Biomedicine, Biotechnology and Public Health, Faculty of Sciences, University of Cádiz, Puerto Real, Spain

†PRESENT ADDRESS

**Valentina Ricciardi**,

Institute of Biosciences and Bioresources, National Research Council, Palermo, Italy

The correct assignment of the affiliations is:

Simona Pizzi^1†^, Alessandra Di Canito^1^, Elena Palencia Mulero^1^, Carmen Cris De Oliveira Nobre Bezerra^1^, Valentina Ricciardi^2†^, Giuliana Maddalena^2^, Evelyn Maluleke^3^, Roberto Carmine Foschino^1^, Silvia Laura Toffolatti^2^, Daniela Fracassetti^4^, Gabriella De Lorenzis^2^, Gustavo Cordero-Bueso^5^, Mathabatha Evodia Setati^3^ and Ileana Vigentini^1*^

^1^Department of Biomedical, Surgical and Dental Sciences (DiSBIOC), Università degli Studi di Milano, Milan, Italy,

^2^Department of Agricultural and Environmental Sciences (DiSAA), Università degli Studi di Milano, Milan, Italy,

^3^South African Grape and Wine Research Institute, Stellenbosch University, Stellenbosch, South Africa,

^4^Department of Food, Environmental and Nutritional Sciences (DeFENS), Università degli Studi di Milano, Milan, Italy,

^5^Department of Biomedicine, Biotechnology and Public Health, Faculty of Sciences, University of Cádiz, Puerto Real, Spain

^†^Present address

Simona Pizzi, Department of Food, Environmental and Nutritional Sciences (DeFENS), Università degli Studi di Milano, Milan, Italy

Valentina Ricciardi, Institute of Biosciences and Bioresources, National Research Council, Palermo, Italy

Additionally, author Simona Pizzi was erroneously assigned to affiliation Department of Food, Environmental and Nutritional Sciences (DeFENS). This affiliation has now been removed for author Simona Pizzi.

The original version of the article has been updated.

