# Supplementary material for: Study of grapevine endophytes’ interaction with the host and their potential as biocontrol agents as sustainable alternative to agrochemicals
Source: Front Microbiol. 2026 Feb 18;17:1758446. doi: 10.3389/fmicb.2026.1758446 (PMC12957226; doi:10.3389/fmicb.2026.1758446)

**Supplementary Materials Figure 1. (A)** Illustration of the experimental tests used to perform the Dual Culture Plate assay (DC) of BCAs against the proliferation of *B. cinerea*.

**
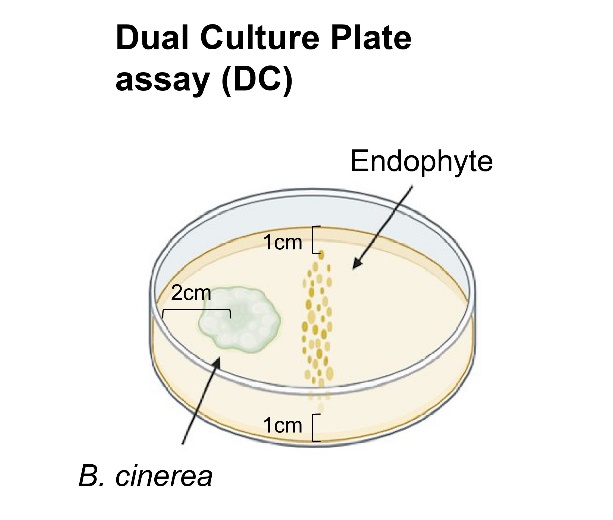
**

**Supplementary Materials Figure 1. (B)** Illustration of the experimental tests used to perform the Double Petri Dish assay (DPD) of BCAs against the proliferation of *B. cinerea*.

**
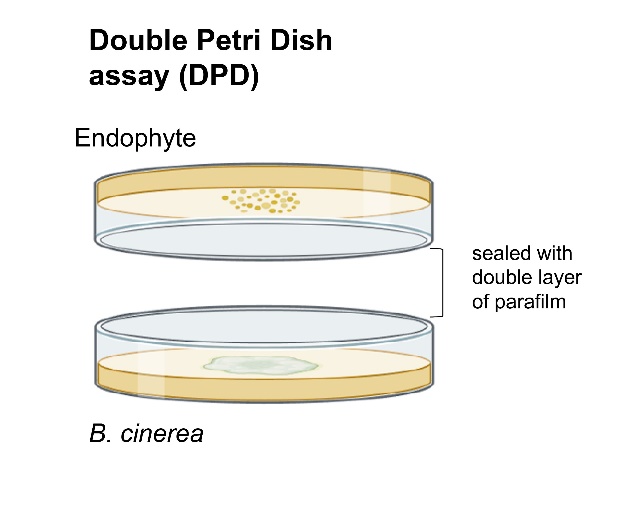
**

**Supplementary Materials Table 1.** List of endophytic isolates tested in the present study.

| Identification | ID CODE | Geografical origin | Plant species | Crop management | Plant organs |
| --- | --- | --- | --- | --- | --- |
| *Aureobasidium pullulans* | ED203 | Franciacorta | V.v. | Abandoned | Shoot |
| *Aureobasidium pullulans* | ED217 | Monte Fenera | V.s. | Wild | Shoot |
| *Aureobasidium pullulans* | ED206 | Franciacorta | V.v. | Abandoned | Shoot |
| *Aureobasidium pullulans* | ED221 | Monte Fenera | V.s. | Wild | Shoot |
| *Bacillus cereus* | ED158 | Franciacorta | V.v. | Conventional IPM | Leaf |
| *Bacillus megaterium* | ED84 | Riccagioia | V.v. | Kamuri Shavi M22E | Leaf |
| *Bacillus velezensis* | ED163 | Franciacorta | V.v. | Conventional IPM | Leaf |
| *Brevibacillus parabrevis* | ED216 | Monte Fenera | V.s. | Wild | Shoot |
| *Pseudomonas extremaustralis* | ED219 | Monte Fenera | V.s. | Wild | Shoot |
| *Curtobacterium flaccumfaciens* | ED207 | Franciacorta | V.v. | Abandoned | Shoot |
| *Curtobacterium flaccumfaciens* | ED52 | Riccagioia | V.v. | Mgaloblishvili L22A | Shoot |
| *Deinococcus citri* | ED140 | Franciacorta | V.v. | Biodynamic | Leaf |
| *Dermacoccus nishinomiyaensis* | ED226 | Monte Fenera | V.s. | Wild | Leaf |
| *Dermacoccus nishinomiyaensis* | ED245 | Franciacorta | V.v. | Conventional IPM | Berry |
| *Enterococcus faecium* | ED230 | Franciacorta | V.v. | Organic | Berry |
| *Filobasidium wieringae* | ED121 | Franciacorta | V.v. | Organic | Leaf |
| *Kocuria rhizophila* | ED256 | Franciacorta | V.v. | Abandoned | Berry |
| *Leifsonia shinshuensis* | ED105 | Riccagioia | V.v. | Kamuri Shavi M22E | Leaf |
| *Leifsonia shinshuensis* | ED161 | Franciacorta | V.v. | Conventional IPM | Leaf |
| *Massilia aurea* | ED53 | Riccagioia | V.v. | Mgaloblishvili L22A | Shoot |
| *Mycobacterium canariasense* | ED119 | Riccagioia | V.v. | Mgaloblishvili L22A | Leaf |
| *Mycobacterium canariasense* | ED167 | Franciacorta | V.v. | Abandoned | Leaf |
| *Mycobacterium canariasense* | ED181 | Monte Fenera | V.s. | Wild | Leaf |
| *Mycobacteroides abscessus* | ED102 | Riccagioia | V.v. | Kamuri Shavi M22E | Leaf |
| *Mycobacteroides abscessus* | ED136 | Franciacorta | V.v. | Organic | Leaf |
| *Mycobacteroides abscessus* | ED190 | Monte Fenera | V.s. | Wild | Leaf |
| *Mycolicibacterium aubagnense* | ED151 | Franciacorta | V.v. | Conventional IPM | Leaf |
| *Nocardia niigatensis* | ED116 | Riccagioia | V.v. | Mgaloblishvili L22A | Leaf |
| *Nocardia niigatensis* | ED127 | Monte Fenera | V.s. | Wild | Leaf |
| *Nocardia niigatensis* | ED186 | Franciacorta | V.v. | Organic | Leaf |
| *Okibacterium fritillariae* | ED58 | Riccagioia | V.v. | Mgaloblishvili L22A | Shoot |
| *Pantoea agglomerans* | ED208 | Monte Fenera | V.s. | Wild | Shoot |
| *Pantoea agglomerans* | ED77 | Riccagioia | V.v. | Kamuri Shavi M22E | Shoot |
| *Paracoccus yeei* | ED74 | Monte Fenera | V.s. | Wild | Shoot |
| *Pseudomonas coleopterorum* | ED232 | Riccagioia | V.v. | Kamuri Shavi M22E | Berry |
| *Ralstonia pickettii* | ED126 | Franciacorta | V.v. | Organic | Leaf |
| *Ralstonia pickettii* | ED179 | Monte Fenera | V.s. | Wild | Leaf |
| *Ralstonia pickettii* | ED82 | Riccagioia | V.v. | Kamuri Shavi M22E | Leaf |
| *Sphingomonas echinoides* | ED120 | Riccagioia | V.v. | Mgaloblishvili L22A | Leaf |
| *Sphingomonas echinoides* | ED132 | Franciacorta | V.v. | Organic | Leaf |
| *Sphingomonas echinoides* | ED180 | Monte Fenera | V.s. | Wild | Leaf |
| *Staphylococcus warneri* | ED244 | Franciacorta | V.v. | Biodynamic | Berry |

**Supplementary Materials Table 2. (A)** List of volatile compounds released by BCAs and *B. cinerea*. (RT) Retention Time, (Rm) Library Match Factor.

| Volatile Organic Compound | Chemical class | RT (min) | R match | Mean ± SD | | | | | |
| --- | --- | --- | --- | --- | --- | --- | --- | --- | --- |
|  |  |  |  | **B05.10** | **ED203** | **ED163** | **ED206** | **ED217** | **ED221** |
| *Acetic acid* | Acid | 18,62 | 834 | 39,88 ± 6,38 | 64,08 ± 21,15 | 284,57 ± 174,97 | 51,48 ± 11,90 | 64,35 ± 14,45 | 81,02 ± 19,49 |
| *Propanoic acid* | Acid | 20,84 | 847 |  | 3,32 ± 0,49 | 0,71 ± 0,22 | 3,42 ± 0,12 | 4,42 ± 1,88 | 2,31 ± 0,34 |
| *Propanoic acid, 2-methyl* | Acid | 21,58 | 840 |  | 16,23 ± 1,88 | 9,48 ± 2,87 | 21,73 ± 3,23 | 12,82 ± 3,18 | 11,68 ± 3,47 |
| *Butanoic acid* | Acid | 27,84 | 881 | 7,72 ± 1,04 |  |  |  |  |  |
| *Octanoic acid* | Acid | 32,16 | 873 | 50,25 ± 12,32 | 17,26 ± 3,75 | 5,20 ± 3,77 | 4,54 ± 0,22 | 2,37 ± 0,16 | 1,17 ± 0,64 |
| *Hexanoic acid* | Acid | 34,17 | 669 | 1,59 ± 0,85 |  |  |  |  |  |
| *Heptanoic acid, 2-ethyl* | Acid | 35,68 | 712 |  | 3,10 ± 1,05 |  | 4,43 ± 1,17 | 14,13 ± 5,08 | 1,19 ± 0,72 |
| *Decanoic acid* | Acid | 36,08 | 624 | 15,27 ± 3,82 | 4,58 ± 1,23 |  | 0,97 ± 0,07 | 0,35 ± 0,05 | 0,36 ± 0,11 |
| *1-Propanol* | Alcohol | 6,48 | 902 |  | 41,88 ± 4,83 |  | 60,94 ± 1,81 | 47,80 ± 16,48 | 25,97 ± 0,72 |
| *1-Propanol, 2-methyl* | Alcohol | 8,47 | 898 |  | 473,55 ± 140,64 | 13,00 ± 5,81 | 559,61 ± 24,98 | 430,67 ± 136,10 | 463,96 ± 196,28 |
| *1-Butanol* | Alcohol | 10,19 | 881 |  | 36,41 ± 10,51 |  | 15,80 ± 2,23 | 8,51 ± 1,36 | 11,11 ± 1,56 |
| *1-Butanol, 3-methyl* | Alcohol | 11,98 | 918 | 13,40 ± 5,17 | 886,73 ± 86,03 | 82,70 ± 32,40 | 1118,94 ± 59,49 | 896,72 ± 121,69 | 1040,30 ± 269,50 |
| *1-Pentanol* | Alcohol | 13,2 | 750 |  | 2,90 ± 0,02 |  | 1,87 ± 0,53 | 2,02 ± 0,85 | 1,08 ± 0,45 |
| *4-Penten-1-ol* | Alcohol | 14,68 | 898 |  | 17,55 ± 4,23 |  | 14,13 ± 3,27 | 18,43 ± 7,14 | 15,37 ± 2,32 |
| *2-Heptanol* | Alcohol | 15,2 | 875 |  | 363,37 ± 54,73 |  | 78,90 ± 1,75 | 337,40 ± 38,59 | 322,13 ± 46,25 |
| *1-Hexanol* | Alcohol | 16,12 | 865 |  | 258,10 ± 51,87 | 92,46 ± 3,11 | 142,23 ± 33,68 | 131,27 ± 26,68 | 89,56 ± 39,02 |
| *dl-6-Dimethyl-5-hepten-2-ol* | Alcohol | 19,04 | 677 |  | 0,76 ± 0,21 |  | 0,59 ± 0,04 |  |  |
| *2,3-Dimethyl-4-penten-2-ol* | Alcohol | 19,16 | 694 |  | 1,18 ± 0,39 |  |  |  |  |
| *1-Hexanol-2-ethyl* | Alcohol | 19,7 | 846 | 30,06 ± 0,12 | 35,75 ± 8,86 | 20,80 ± 6,95 | 28,92 ± 7,80 | 42,79 ± 15,29 | 26,87 ± 7,30 |
| *2-Nonanol* | Alcohol | 20,46 | 906 |  | 247,77 ± 10,18 |  | 176,97 ± 25,60 | 307,31 ± 63,71 | 253,58 ± 78,74 |
| *1-Octanol* | Alcohol | 21,42 | 836 |  | 33,67 ± 8,33 | 1,09 ± 0,64 | 66,72 ± 2,96 | 35,23 ± 2,55 | 19,74 ± 8,06 |
| *2-Furanmethanol* | Alcohol | 23,82 | 897 |  | 156,75 ± 19,55 |  | 107,59 ± 5,27 | 119,77 ± 9,37 | 100,74 ± 16,32 |
| *Phenylethyl alcohol* | Alcohol | 29,27 | 940 |  | 1531,13 ± 326,09 | 129,46 ± 76,78 | 1867,08 ± 476,63 | 1181,04 ± 217,15 | 1250,62 ± 390,88 |
| *Butanal, 3-methyl* | Aldehyde | 3,32 | 830 | 22,42 ± 4,93 | 21,85 ± 6,11 |  | 22,52 ± 18,23 | 4,10 ± 1,29 | 39,21 ± 34,99 |
| *Furfural* | Aldehyde | 18,86 | 936 | 13,17 ± 11,32 |  |  |  |  |  |
| *Benzaldehyde* | Aldehyde | 20,28 | 858 | 29,72 ± 24,79 |  |  |  |  |  |
| *Benzeneacetaldehyde* | Aldehyde | 23,17 | 870 | 7,41 ± 0,07 |  |  |  |  |  |
| *Hexane, 3,3-dimethyl* | Alkane | 6,04 | 839 | 13,84 ± 2,14 | 20,97 ± 3,67 | 63,54 ± 0,99 | 19,75 ± 6,67 | 33,10 ± 3,42 | 22,12 ± 2,81 |
| *Undecane, 4,7.dimethyl* | Alkane | 7,65 | 794 | 3,96 ± 0,32 | 3,30 ± 0,14 | 7,23 ± 3,06 | 2,82 ± 0,93 | 4,60 ± 0,34 | 2,56 ± 0,48 |
| *Hexadecane* | Alkane | 12,34 | 804 | 78,46 ± 14,02 | 42,84 ± 8,87 | 53,83 ± 40,91 | 39,66 ± 13,20 | 39,78 ± 5,18 | 55,43 ± 12,61 |
| *3-Ethyl-3-methylheptane* | Alkane | 17,96 | 735 | 1,37 ± 0,65 |  |  |  |  |  |
| *Undecane, 3,8-dimethyl* | Alkane | 18,1 | 840 | 41,02 ± 7,42 |  |  |  |  |  |
| *Octane, 3,4,5,6-tetramethyl* | Alkane | 18,36 | 730 | 2,52 ± 1,60 | 2,94 ± 0,88 |  | 4,58 ± 1,48 | 4,04 ± 0,04 | 5,00 ± 2,15 |
| *Bicyclo[3.1.0]hexane, 4-methylene-1-(1methylethyl)* | Alkane | 21,1 | 743 |  | 2,09 ± 0,55 |  | 15,44 ± 0,36 | 2,30 ± 0,90 | 1,36 ± 0,52 |
| *2-Pentene, 5-butoxy, (E)* | Alkene | 5,3 | 846 |  | 46,11 ± 2,60 |  |  |  |  |
| *1-Nonene, 4,6,8-trimethyl* | Alkene | 14,15 | 713 | 3,10 ± 0,84 |  |  |  |  |  |
| *4-Octene, 2,3,7,-trimethyl [S-(E)]* | Alkene | 14,48 | 788 | 4,35 ± 1,95 |  |  |  |  |  |
| *2,4,6,8-Tetramethyl-1-u0ecene* | Alkene | 14,74 | 699 | 1,20 ± 0,37 |  |  |  |  |  |
| *Cyclohexene, 1-methyl-3-(1-methylethyl)* | Alkene | 21,71 | 722 |  | 10,80 ± 1,43 |  | 1,68 ± 0,02 | 1,50 ± 0,43 | 1,67 ± 0,38 |
| *Benzene, (3-chloro-1-methyl-1-propenyl)* | Alkene | 26,84 | 845 |  | 233,33 ± 19,98 | 156,40 ± 74,56 | 187,74 ± 18,35 | 268,88 ± 3,83 | 181,33 ± 43,87 |
| *Bicyclo(3.2.0)hepta-2,6-diene* | Alkene | 29,27 | 901 | 4,43 ± 1,44 |  |  |  |  |  |
| *Formamide, N,N-dimethyl* | Amide | 33,38 | 717 |  |  |  |  |  |  |
| *Ethyl acetate* | Ester | 2,94 | 872 | 19,56 ± 15,79 | 142,21 ± 50,74 | 21,23 ± 0,85 | 148,36 ± 27,30 | 84,75 ± 26,75 | 160,90 ± 8,44 |
| *Acetic acid, 2-methylpropyl ester* | Ester | 5,45 | 821 |  | 21,75 ± 7,20 |  | 15,33 ± 3,31 |  | 20,57 ± 1,39 |
| *Butanoic acid, ethyl ester* | Ester | 6,17 | 849 | 2,94 ± 1,79 | 12,13 ± 2,98 |  | 8,13 ± 1,08 | 10,59 ± 1,98 | 7,67 ± 1,41 |
| *Oxalic acid, isobutyl nonyl ester* | Ester | 7,49 | 799 | 12,81 ± 8,72 | 17,01 ± 1,68 | 39,87 ± 24,54 | 13,49 ± 2,44 | 19,74 ± 0,69 | 12,88 ± 1,27 |
| *Sulfurous acid, 2-ethylhexyl hexyl ester* | Ester | 12,42 | 744 | 6,97 ± 1,42 |  |  |  |  |  |
| *Sulfurous acid, 2-ethylhexyl isohexyl ester* | Ester | 13,99 | 794 | 25,99 ± 8,80 | 7,33 ± 0,00 | 25,77 ± 0,86 | 4,55 ± 0,76 | 14,95 ± 1,19 | 2,44 ± 0,42 |
| *2-Acetoxydodecane* | Ester | 27,89 | 765 |  | 86,04 ± 9,85 |  | 22,61 ± 0,86 | 73,94 ± 21,99 | 58,03 ± 14,86 |
| *Propanoic acid, 2-methyl, 3-hydroxy-2,4,4, trimethylpenthyl ester* | Ester | 28,28 | 794 | 22,40 ± 15,28 |  |  |  |  |  |
| *2-Furanmethanol, tetrahydro acetate* | Ester | 28,61 | 806 | 7,38 ± 5,79 |  |  |  |  |  |
| *Propanoic acid, ethylen ester* | Ester | 36,78 | 749 | 11,79 ± 4,24 | 1,50 ± 0,36 | 8,83 ± 3,46 |  |  | 0,38 ± 0,03 |
| *p-Anisic acid, 4-nitrophenyl ester* | Ester | 38,42 | 849 |  | 1,03 ± 0,59 | 1,86 ± 0,45 | 0,87 ± 0,20 | 0,83 ± 0,27 | 0,73 ± 0,32 |
| *Oxirane, 2,3-dimethyl* | Ether | 4,49 | 897 | 1,10 ± 0,74 |  |  |  |  |  |
| *Ether, 3-butenyl pentyl* | Ether | 22,94 | 730 | 2,92 ± 1,25 |  |  |  |  |  |
| *2-Pentanone* | Ketone | 4,45 | 814 |  | 54,53 ± 0,48 |  |  |  |  |
| *2-Heptanone* | Ketone | 10,65 | 907 | 4,33 ± 2,48 | 347,42 ± 61,15 |  | 89,46 ± 5,49 | 221,05 ± 8,22 | 185,45 ± 42,87 |
| *4-Heptanone, 3-methyl* | Ketone | 12,62 | 744 | 3,29 ± 1,14 |  |  |  |  |  |
| *3-Hexanone, 2,5-dimethyl* | Ketone | 12,84 | 782 | 3,82 ± 1,03 |  |  |  |  |  |
| *2-Butanone, 3-hydroxy* | Ketone | 14,06 | 879 |  | 16,24 ± 5,18 | 12,96 ± 5,37 | 10,90 ± 1,33 |  |  |
| *2-Nonanone* | Ketone | 16,82 | 882 | 5,64 ± 3,47 | 185,08 ± 13,97 | 21,99 ± 21,36 | 116,89 ± 30,90 | 107,28 ± 14,57 | 101,10 ± 33,30 |
| *2-Cyclopentene-1,4-dione* | Ketone | 21,86 | 750 | 3,45 ± 1,76 |  |  |  |  |  |
| *1-Bicyclo(3,3,1)non-6-en-3-yl-2-methylpropan-1-one* | Ketone | 24,63 | 624 |  | 7,80 ± 2,07 |  | 7,31 ± 0,61 | 12,96 ± 2,63 | 8,87 ± 2,63 |
| *Ethanone, 1-(1H-pyrrol-2-yl)* | Ketone | 30,44 | 781 | 17,36 ± 1,74 |  |  |  |  |  |
| *Propionolactone* | Lactone | 14,55 | 909 | 2,47 ± 1,23 |  |  |  |  |  |
| *4-Pyridazinamine* | N-heterocycle | 12,6 | 677 |  | 9,46 ± 0,84 |  | 10,28 ± 2,88 | 13,22 ± 4,45 |  |
| *Pyrazine, 2,6-diethyl* | N-heterocycle | 18,46 | 898 | 2,89 ± 0,24 |  |  |  |  |  |
| *1H-Imidazole, 1,5-dimethyl* | N-heterocycle | 18,85 | 622 |  | 3,89 ± 1,30 | 400,01 ± 22,17 |  |  |  |
| *Indole* | N-heterocycle | 38,98 | 809 |  | 0,86 ± 0,03 |  | 0,37 ± 0,16 | 3,90 ± 0,87 | 0,31 ± 0,29 |
| *2-Buten-1-one, 1-(2,6,6-trinethyl-1,3-cyclohexadien-1-yl), (E)* | Terpene | 27,26 | 774 |  | 18,13 ± 1,63 |  | 26,00 ± 7,74 | 13,91 ± 4,73 | 7,86 ± 2,86 |

**Supplementary Materials Table 2. (B)** List of volatile compounds released by the interaction BCA-pathogen. (RT) Retention Time, (Rm) Library Match Factor.

| Volatile Organic Compound | Chemical class | RT (min) | R match | Mean ± SD | | | | |
| --- | --- | --- | --- | --- | --- | --- | --- | --- |
|  |  |  |  | **ED203 + B05.10** | **ED163 + B05.10** | **ED206 + B05.10** | **ED217 + ED05.10** | **ED221 + B05.10** |
| *Acetic acid* | Acid | 18,62 | 834 | 82,03 ± 3,35 | 158,69 ± 25,84 | 71,97 ± 15,60 | 64,05 ± 18,66 | 47,05 ± 4,26 |
| *Propanoic acid* | Acid | 20,84 | 847 | 3,02 ± 0,36 | 0,51 ± 0,17 | 3,45 ± 1,18 | 3,70 ± 1,00 | 3,14 ± 0,34 |
| *Propanoic acid, 2-methyl* | Acid | 21,58 | 840 | 14,21 ± 1,68 | 2,78 ± 0,48 | 21,66 ± 3,87 | 9,94 ± 1,72 | 11,30 ± 1,24 |
| *Butanoic acid* | Acid | 27,84 | 881 |  |  |  |  |  |
| *Octanoic acid* | Acid | 32,16 | 873 | 10,40 ± 4,21 | 3,34 ± 0,85 | 3,14 ± 0,41 | 1,75 ± 0,37 | 0,60 ± 0,10 |
| *Hexanoic acid* | Acid | 34,17 | 669 |  |  |  |  |  |
| *Heptanoic acid, 2-ethyl* | Acid | 35,68 | 712 | 16,68 ± 3,83 |  | 7,12 ± 2,81 | 3,06 ± 1,39 | 9,86 ± 2,61 |
| *Decanoic acid* | Acid | 36,08 | 624 | 2,24 ± 0,76 |  |  | 0,30 ± 0,13 |  |
| *1-Propanol* | Alcohol | 6,48 | 902 | 41,63 ± 10,00 |  | 54,92 ± 5,29 | 33,03 ± 4,91 | 37,90 ± 6,51 |
| *1-Propanol, 2-methyl* | Alcohol | 8,47 | 898 | 383,83 ± 45,95 | 6,08 ± 1,95 | 549,51 ± 44,58 | 359,21 ± 34,62 | 323,27 ± 35,12 |
| *1-Butanol* | Alcohol | 10,19 | 881 | 28,15 ± 2,38 |  | 12,32 ± 0,70 | 8,31 ± 1,70 | 11,14 ± 0,95 |
| *1-Butanol, 3-methyl* | Alcohol | 11,98 | 918 | 800,93 ± 45,77 | 48,51 ± 3,58 | 1067,33 ± 35,54 | 867,46 ± 153,67 | 789,67 ± 133,06 |
| *1-Pentanol* | Alcohol | 13,2 | 750 | 3,30 ± 0,36 |  | 1,84 ± 0,68 | 2,27 ± 0,08 | 2,39 ± 1,14 |
| *4-Penten-1-ol* | Alcohol | 14,68 | 898 | 12,78 ± 2,43 |  | 13,21 ± 2,56 | 16,64 ± 5,81 | 16,62 ± 4,45 |
| *2-Heptanol* | Alcohol | 15,2 | 875 | 459,72 ± 117,78 |  | 68,53 ± 8,77 | 332,99 ± 39,68 | 225,63 ± 79,25 |
| *1-Hexanol* | Alcohol | 16,12 | 865 | 228,69 ± 17,72 | 4,97 ± 2,24 | 149,65 ± 49,63 | 93,00 ± 29,73 | 164,52 ± 32,22 |
| *dl-6-Dimethyl-5-hepten-2-ol* | Alcohol | 19,04 | 677 | 0,58 ± 0,10 |  |  |  |  |
| *2,3-Dimethyl-4-penten-2-ol* | Alcohol | 19,16 | 694 | 0,93 ± 0,20 |  |  |  |  |
| *1-Hexanol-2-ethyl* | Alcohol | 19,7 | 846 | 27,93 ± 2,43 | 7,47 ± 3,09 | 32,93 ± 2,05 | 30,38 ± 4,08 | 28,05 ± 2,65 |
| *2-Nonanol* | Alcohol | 20,46 | 906 | 376,18 ± 53,15 |  | 167,27 ± 6,57 | 264,19 ± 153,09 | 269,42 ± 61,77 |
| *1-Octanol* | Alcohol | 21,42 | 836 | 37,67 ± 2,23 |  | 47,01 ± 22,50 | 30,21 ± 8,16 | 23,28 ± 12,90 |
| *2-Furanmethanol* | Alcohol | 23,82 | 897 | 101,58 ± 1,46 | 60,98 ± 8,20 | 128,73 ± 18,20 | 117,13 ± 11,93 | 118,98 ± 8,71 |
| *Phenylethyl alcohol* | Alcohol | 29,27 | 940 | 1257,05 ± 162,79 | 88,43 ± 15,53 | 1512,57 ± 51,61 | 1049,56 ± 215,45 | 1084,70 ± 79,52 |
| *Butanal, 3-methyl* | Aldehyde | 3,32 | 830 | 10,96 ± 3,49 | 7,20 ± 0,52 | 15,21 ± 4,35 | 5,31 ± 2,01 | 5,28 ± 0,19 |
| *Furfural* | Aldehyde | 18,86 | 936 |  |  |  |  |  |
| *Benzaldehyde* | Aldehyde | 20,28 | 858 |  |  |  |  |  |
| *Benzeneacetaldehyde* | Aldehyde | 23,17 | 870 |  |  |  |  |  |
| *Hexane, 3,3-dimethyl* | Alkane | 6,04 | 839 | 30,91 ± 4,36 | 26,18 ± 0,79 | 31,13 ± 2,79 | 35,87 ± 6,15 | 37,26 ± 3,62 |
| *Undecane, 4,7.dimethyl* | Alkane | 7,65 | 794 | 5,28 ± 1,08 | 2,80 ± 0,12 | 5,05 ± 0,59 | 5,49 ± 0,48 | 5,37 ± 0,71 |
| *Hexadecane* | Alkane | 12,34 | 804 | 61,78 ± 3,01 | 84,15 ± 13,84 | 54,60 ± 17,54 | 82,36 ± 10,77 | 44,48 ± 9,01 |
| *3-Ethyl-3-methylheptane* | Alkane | 17,96 | 735 |  |  |  |  |  |
| *Undecane, 3,8-dimethyl* | Alkane | 18,1 | 840 |  |  |  |  |  |
| *Octane, 3,4,5,6-tetramethyl* | Alkane | 18,36 | 730 | 5,90 ± 1,58 |  | 4,19 ± 1,16 | 4,84 ± 1,88 | 4,26 ± 2,24 |
| *Bicyclo[3.1.0]hexane, 4-methylene-1-(1methylethyl)* | Alkane | 21,1 | 743 | 1,26 ± 0,33 |  | 10,45 ± 1,25 | 1,50 ± 0,51 | 1,21 ± 0,23 |
| *2-Pentene, 5-butoxy, (E)* | Alkene | 5,3 | 846 | 41,59 ± 2,79 | 16,82 ± 9,41 |  |  |  |
| *1-Nonene, 4,6,8-trimethyl* | Alkene | 14,15 | 713 |  |  |  |  |  |
| *4-Octene, 2,3,7,-trimethyl [S-(E)]* | Alkene | 14,48 | 788 |  |  |  |  |  |
| *2,4,6,8-Tetramethyl-1-u0ecene* | Alkene | 14,74 | 699 |  |  |  |  |  |
| *Cyclohexene, 1-methyl-3-(1-methylethyl)* | Alkene | 21,71 | 722 | 1,64 ± 0,27 |  | 1,20 ± 0,19 | 1,38 ± 0,29 | 2,51 ± 0,97 |
| *Benzene, (3-chloro-1-methyl-1-propenyl)* | Alkene | 26,84 | 845 | 150,89 ± 38,22 | 29,38 ± 10,33 | 256,25 ± 23,78 | 213,12 ± 30,92 | 199,14 ± 27,76 |
| *Bicyclo(3.2.0)hepta-2,6-diene* | Alkene | 29,27 | 901 |  |  |  |  |  |
| *Formamide, N,N-dimethyl* | Amide | 33,38 | 717 |  |  |  |  |  |
| *Ethyl acetate* | Ester | 2,94 | 872 | 92,72 ± 8,12 |  | 91,12 ± 10,57 | 76,71 ± 7,44 | 151,86 ± 12,11 |
| *Acetic acid, 2-methylpropyl ester* | Ester | 5,45 | 821 | 12,70 ± 1,51 |  | 12,23 ± 1,50 |  | 16,36 ± 3,62 |
| *Butanoic acid, ethyl ester* | Ester | 6,17 | 849 | 14,91 ± 1,17 |  | 10,87 ± 0,18 | 10,36 ± 0,91 | 11,64 ± 1,55 |
| *Oxalic acid, isobutyl nonyl ester* | Ester | 7,49 | 799 | 22,89 ± 3,73 | 19,05 ± 3,47 | 20,43 ± 0,89 | 22,37 ± 1,50 | 21,34 ± 1,74 |
| *Sulfurous acid, 2-ethylhexyl hexyl ester* | Ester | 12,42 | 744 |  |  |  |  |  |
| *Sulfurous acid, 2-ethylhexyl isohexyl ester* | Ester | 13,99 | 794 | 20,32 ± 1,86 | 15,86 ± 9,08 | 20,84 ± 6,10 | 21,87 ± 3,15 | 13,96 ± 2,08 |
| *2-Acetoxydodecane* | Ester | 27,89 | 765 | 121,46 ± 3,09 |  | 7,25 ± 1,86 | 80,52 ± 11,18 | 34,86 ± 6,00 |
| *Propanoic acid, 2-methyl, 3-hydroxy-2,4,4, trimethylpenthyl ester* | Ester | 28,28 | 794 |  |  |  |  |  |
| *2-Furanmethanol, tetrahydro acetate* | Ester | 28,61 | 806 |  |  |  |  |  |
| *Propanoic acid, ethylen ester* | Ester | 36,78 | 749 | 1,07 ± 0,34 | 1,24 ± 0,45 |  |  |  |
| *p-Anisic acid, 4-nitrophenyl ester* | Ester | 38,42 | 849 | 0,84 ± 0,17 | 0,72 ± 0,17 | 0,90 ± 0,32 | 0,70 ± 0,36 | 0,70 ± 0,38 |
| *Oxirane, 2,3-dimethyl* | Ether | 4,49 | 897 |  |  |  |  |  |
| *Ether, 3-butenyl pentyl* | Ether | 22,94 | 730 |  |  |  |  |  |
| *2-Pentanone* | Ketone | 4,45 | 814 | 66,46 ± 15,35 |  |  |  |  |
| *2-Heptanone* | Ketone | 10,65 | 907 | 513,90 ± 33,32 |  | 104,23 ± 24,91 | 250,95 ± 21,26 | 187,23 ± 12,64 |
| *4-Heptanone, 3-methyl* | Ketone | 12,62 | 744 |  |  |  |  |  |
| *3-Hexanone, 2,5-dimethyl* | Ketone | 12,84 | 782 |  |  |  |  |  |
| *2-Butanone, 3-hydroxy* | Ketone | 14,06 | 879 | 5,71 ± 0,01 | 16,55 ± 9,02 |  |  |  |
| *2-Nonanone* | Ketone | 16,82 | 882 | 281,29 ± 40,25 |  | 194,91 ± 28,49 | 47,45 ± 33,49 | 122,73 ± 22,64 |
| *2-Cyclopentene-1,4-dione* | Ketone | 21,86 | 750 |  |  |  |  |  |
| *1-Bicyclo(3,3,1)non-6-en-3-yl-2-methylpropan-1-one* | Ketone | 24,63 | 624 | 5,53 ± 2,33 |  | 9,16 ± 2,46 | 8,03 ± 4,38 | 11,17 ± 3,01 |
| *Ethanone, 1-(1H-pyrrol-2-yl)* | Ketone | 30,44 | 781 |  |  |  |  |  |
| *Propionolactone* | Lactone | 14,55 | 909 |  |  |  |  |  |
| *4-Pyridazinamine* | N-heterocycle | 12,6 | 677 | 8,98 ± 2,28 |  | 12,02 ± 1,12 | 11,09 ± 2,59 |  |
| *Pyrazine, 2,6-diethyl* | N-heterocycle | 18,46 | 898 |  |  |  |  |  |
| *1H-Imidazole, 1,5-dimethyl* | N-heterocycle | 18,85 | 622 | 2,19 ± 0,61 | 35,04 ± 19,38 |  |  |  |
| *Indole* | N-heterocycle | 38,98 | 809 | 0,59 ± 0,18 |  | 1,65 ± 1,21 | 4,17 ± 2,17 | 0,97 ± 0,59 |
| *2-Buten-1-one, 1-(2,6,6-trinethyl-1,3-cyclohexadien-1-yl), (E)* | Terpene | 27,26 | 774 | 14,57 ± 5,97 |  | 25,39 ± 3,15 | 9,18 ± 0,94 | 9,87 ± 3,31± |

**Supplementary Materials Table 2. (C)** List of volatile compounds released by the interaction BCA-pathogen. Data are expressed as difference of the response factor (x10^4^) between the trial with co-culture, with BCA and by *B. cinerea* alone. A positive value indicates compounds produced in higher amounts during the interaction. (RT) Retention Time, (Rm) Library Match Factor.

| Volatile Organic Compound | Chemical class | RT (min) | R match | Difference | | | | |
| --- | --- | --- | --- | --- | --- | --- | --- | --- |
|  |  |  |  | **ED203 + B05.10** | **ED163 + B05.10** | **ED206 + B05.10** | **ED217 + ED05.10** | **ED221 + B05.10** |
| *Acetic acid* | Acid | 18,62 | 834 | -21,93 | -165,76 | -19,39 | -40,18 | -73,84 |
| *Butanoic acid* | Acid | 27,84 | 881 | -7,72 | -7,72 | -7,72 | -7,72 | -7,72 |
| *Decanoic acid* | Acid | 36,08 | 624 | -17,61 | -15,27 | -16,24 | -15,32 | -15,63 |
| *Heptanoic acid, 2-ethyl* | Acid | 35,68 | 712 | 13,57 |  | 2,69 | -11,06 | 8,68 |
| *Hexanoic acid* | Acid | 34,17 | 669 | -1,59 | -1,59 | -1,59 | -1,59 | -1,59 |
| *Octanoic acid* | Acid | 32,16 | 873 | -57,12 | -52,11 | -51,66 | -50,87 | -50,82 |
| *Propanoic acid* | Acid | 20,84 | 847 | -0,30 | -0,20 | 0,03 | -0,72 | 0,83 |
| *Propanoic acid, 2-methyl* | Acid | 21,58 | 840 | -2,02 | -6,70 | -0,07 | -2,88 | -0,39 |
| *1-Butanol* | Alcohol | 10,19 | 881 | -8,26 |  | -3,49 | -0,21 | 0,04 |
| *1-Butanol, 3-methyl* | Alcohol | 11,98 | 918 | -99,19 | -47,59 | -65,00 | -42,66 | -264,02 |
| *1-Hexanol* | Alcohol | 16,12 | 865 | -29,41 | -87,49 | 7,41 | -38,26 | 74,97 |
| *1-Hexanol-2-ethyl* | Alcohol | 19,7 | 846 | -37,88 | -43,40 | -26,05 | -42,48 | -28,89 |
| *1-Octanol* | Alcohol | 21,42 | 836 | 4,00 | -1,09 | -19,71 | -5,02 | 3,54 |
| *1-Pentanol* | Alcohol | 13,2 | 750 | 0,40 |  | -0,03 | 0,25 | 1,32 |
| *1-Propanol* | Alcohol | 6,48 | 902 | -0,25 |  | -6,02 | -14,77 | 11,92 |
| *1-Propanol, 2-methyl* | Alcohol | 8,47 | 898 | -89,72 | -6,92 | -10,10 | -71,46 | -140,69 |
| *2,3-Dimethyl-4-penten-2-ol* | Alcohol | 19,16 | 694 | -0,25 |  |  |  |  |
| *2-Furanmethanol* | Alcohol | 23,82 | 897 | -55,18 | 60,98 | 21,14 | -2,64 | 18,24 |
| *2-Heptanol* | Alcohol | 15,2 | 875 | 96,35 |  | -10,36 | -4,41 | -96,49 |
| *2-Nonanol* | Alcohol | 20,46 | 906 | 128,41 |  | -9,71 | -43,12 | 15,84 |
| *4-Penten-1-ol* | Alcohol | 14,68 | 898 | -4,77 |  | -0,92 | -1,78 | 1,25 |
| *dl-6-Dimethyl-5-hepten-2-ol* | Alcohol | 19,04 | 677 | -0,17 |  | -0,59 |  |  |
| *Phenylethyl alcohol* | Alcohol | 29,27 | 940 | -274,08 | -41,03 | -354,51 | -131,48 | -165,92 |
| *Benzaldehyde* | Aldehyde | 20,28 | 858 | -29,72 | -29,72 | -29,72 | -29,72 | -29,72 |
| *Benzeneacetaldehyde* | Aldehyde | 23,17 | 870 | -7,41 | -7,41 | -7,41 | -7,41 | -7,41 |
| *Butanal, 3-methyl* | Aldehyde | 3,32 | 830 | -33,31 | -15,21 | -29,72 | -21,20 | -56,35 |
| *Furfural* | Aldehyde | 18,86 | 936 | -13,17 | -13,17 | -13,17 | -13,17 | -13,17 |
| *3-Ethyl-3-methylheptane* | Alkane | 17,96 | 735 | -1,37 | -1,37 | -1,37 | -1,37 | -1,37 |
| *Bicyclo[3.1.0]hexane, 4-methylene-1-(1methylethyl)/sabinene (monoterpene)* | Alkane | 21,1 | 743 | -0,83 |  | -5,00 | -0,80 | -0,15 |
| *Hexadecane* | Alkane | 12,34 | 804 | -59,53 | -48,14 | -63,52 | -35,88 | -89,41 |
| *Hexane, 3,3-dimethyl* | Alkane | 6,04 | 839 | -3,90 | -51,20 | -2,46 | -11,06 | 1,31 |
| *Octane, 3,4,5,6-tetramethyl* | Alkane | 18,36 | 730 | 2,96 |  | -0,38 | 0,80 | -0,74 |
| *Undecane, 3,8-dimethyl* | Alkane | 18,1 | 840 | -41,02 | -41,02 | -41,02 | -41,02 | -41,02 |
| *Undecane, 4,7.dimethyl* | Alkane | 7,65 | 794 | -1,98 | -8,38 | -1,73 | -3,06 | -1,15 |
| *1-Nonene, 4,6,8-trimethyl* | Alkene | 14,15 | 713 | -3,10 | -3,10 | -3,10 | -3,10 | -3,10 |
| *2,4,6,8-Tetramethyl-1-u0ecene* | Alkene | 14,74 | 699 | -1,20 | -1,20 | -1,20 | -1,20 | -1,20 |
| *2-Pentene, 5-butoxy, (E)* | Alkene | 5,3 | 846 | -4,52 | 16,82 |  |  |  |
| *4-Octene, 2,3,7,-trimethyl [S-(E)]* | Alkene | 14,48 | 788 | -4,35 | -4,35 | -4,35 | -4,35 | -4,35 |
| *Benzene, (3-chloro-1-methyl-1-propenyl)* | Alkene | 26,84 | 845 | -82,44 | -127,02 | 68,50 | -55,76 | 17,81 |
| *Bicyclo(3.2.0)hepta-2,6-diene* | Alkene | 29,27 | 901 | -4,43 | -4,43 | -4,43 | -4,43 | -4,43 |
| *Cyclohexene, 1-methyl-3-(1-methylethyl)* | Alkene | 21,71 | 722 | -9,16 |  | -0,48 | -0,12 | 0,84 |
| *Formamide, N,N-dimethyl* | Amide | 33,38 | 717 |  |  |  |  |  |
| *2-Acetoxydodecane* | Ester | 27,89 | 765 | 35,42 |  | -15,36 | 6,58 | -23,18 |
| *2-Furanmethanol, tetrahydro acetate* | Ester | 28,61 | 806 | -7,38 | -7,38 | -7,38 | -7,38 | -7,38 |
| *Acetic acid, 2-methylpropyl ester* | Ester | 5,45 | 821 | -9,05 |  | -3,11 |  | -4,21 |
| *Butanoic acid, ethyl ester* | Ester | 6,17 | 849 | -0,15 | -2,94 | -0,20 | -3,17 | 1,03 |
| *Ethyl acetate* | Ester | 2,94 | 872 | -69,05 | -40,79 | -76,80 | -27,59 | -28,59 |
| *Oxalic acid, isobutyl nonyl ester* | Ester | 7,49 | 799 | -6,94 | -33,63 | -5,87 | -10,18 | -4,35 |
| *p-Anisic acid, 4-nitrophenyl ester* | Ester | 38,42 | 849 | -0,19 | -1,15 | 0,03 | -0,13 | -0,03 |
| *Propanoic acid, 2-methyl, 3-hydroxy-2,4,4, trimethylpenthyl ester* | Ester | 28,28 | 794 | -22,40 | -22,40 | -22,40 | -22,40 | -22,40 |
| *Propanoic acid, ethylen ester* | Ester | 36,78 | 749 | -12,23 | -19,38 | -11,79 | -11,79 | -12,17 |
| *Sulfurous acid, 2-ethylhexyl hexyl ester* | Ester | 12,42 | 744 | -6,97 | -6,97 | -6,97 | -6,97 | -6,97 |
| *Sulfurous acid, 2-ethylhexyl isohexyl ester* | Ester | 13,99 | 794 | -13,01 | -35,90 | -9,70 | -19,08 | -14,47 |
| *Ether, 3-butenyl pentyl* | Ether | 22,94 | 730 | -2,92 | -2,92 | -2,92 | -2,92 | -2,92 |
| *Oxirane, 2,3-dimethyl* | Ether | 4,49 | 897 | -1,10 | -1,10 | -1,10 | -1,10 | -1,10 |
| *1-Bicyclo(3,3,1)non-6-en-3-yl-2-methylpropan-1-one* | Ketone | 24,63 | 624 | -2,27 |  | 1,85 | -4,93 | 2,31 |
| *2-Butanone, 3-hydroxy* | Ketone | 14,06 | 879 | -10,53 | 3,59 | -10,90 |  |  |
| *2-Cyclopentene-1,4-dione* | Ketone | 21,86 | 750 | -3,45 | -3,45 | -3,45 | -3,45 | -3,45 |
| *2-Heptanone* | Ketone | 10,65 | 907 | 162,14 | -4,33 | 10,43 | 25,56 | -2,56 |
| *2-Nonanone* | Ketone | 16,82 | 882 | 90,57 | -27,62 | 72,39 | -65,47 | 16,00 |
| *2-Pentanone* | Ketone | 4,45 | 814 | 11,93 |  |  |  |  |
| *3-Hexanone, 2,5-dimethyl* | Ketone | 12,84 | 782 | -3,82 | -3,82 | -3,82 | -3,82 | -3,82 |
| *4-Heptanone, 3-methyl* | Ketone | 12,62 | 744 | -3,29 | -3,29 | -3,29 | -3,29 | -3,29 |
| *Ethanone, 1-(1H-pyrrol-2-yl)* | Ketone | 30,44 | 781 | -17,36 | -17,36 | -17,36 | -17,36 | -17,36 |
| *Propionolactone* | Lactone | 14,55 | 909 | -2,47 | -2,47 | -2,47 | -2,47 | -2,47 |
| *1H-Imidazole, 1,5-dimethyl* | N-heterocycle | 18,85 | 622 | -1,70 | -364,97 |  |  |  |
| *4-Pyridazinamine* | N-heterocycle | 12,6 | 677 | -0,49 |  | 1,73 | -2,13 |  |
| *Idole* | N-heterocycle | 38,98 | 809 | -0,27 |  | 1,28 | 0,27 | 0,67 |
| *Pyrazine, 2,6-diethyl* | N-heterocycle | 18,46 | 898 | -2,89 | -2,89 | -2,89 | -2,89 | -2,89 |
| *2-Buten-1-one, 1-(2,6,6-trinethyl-1,3-cyclohexadien-1-yl), (E)/(E)-β-damascenone* | Terpene | 27,26 | 774 | -3,56 |  | -0,61 | -4,73 | 2,01 |

**Supplementary materials Table 3.** ANOVA. Analysis of Variance for value. The table reports the main effects of the factors included in the model:
(i) *Phenylpropanoid pathway genes* (STS1 vs. VvABCG44),
(ii) *Days after incubation* (4 vs. 8 days), and
(iii) *presence/absence of the BCA* in co-culture.

| Main effects | Sum of squares | Df | Mean Square | F-Ratio | P-value |
| --- | --- | --- | --- | --- | --- |
| Phenylpropanoid pathway genes | 314.76 | 1 | 314.76 | 6.44 | 0.0149 |
| Days after incubation | 3.55376 | 1 | 3.55376 | 0.07 | 0,7887 |
| BCA presence/absence | 1179.18 | 1 | 1179.18 | 24.13 | 0.000 |

**Supplementary materials Figure 2.** ANOVA. The figure shows the mean expression values and the 95% confidence intervals obtained from Tukey’s HSD test for the factors included in the ANOVA model.


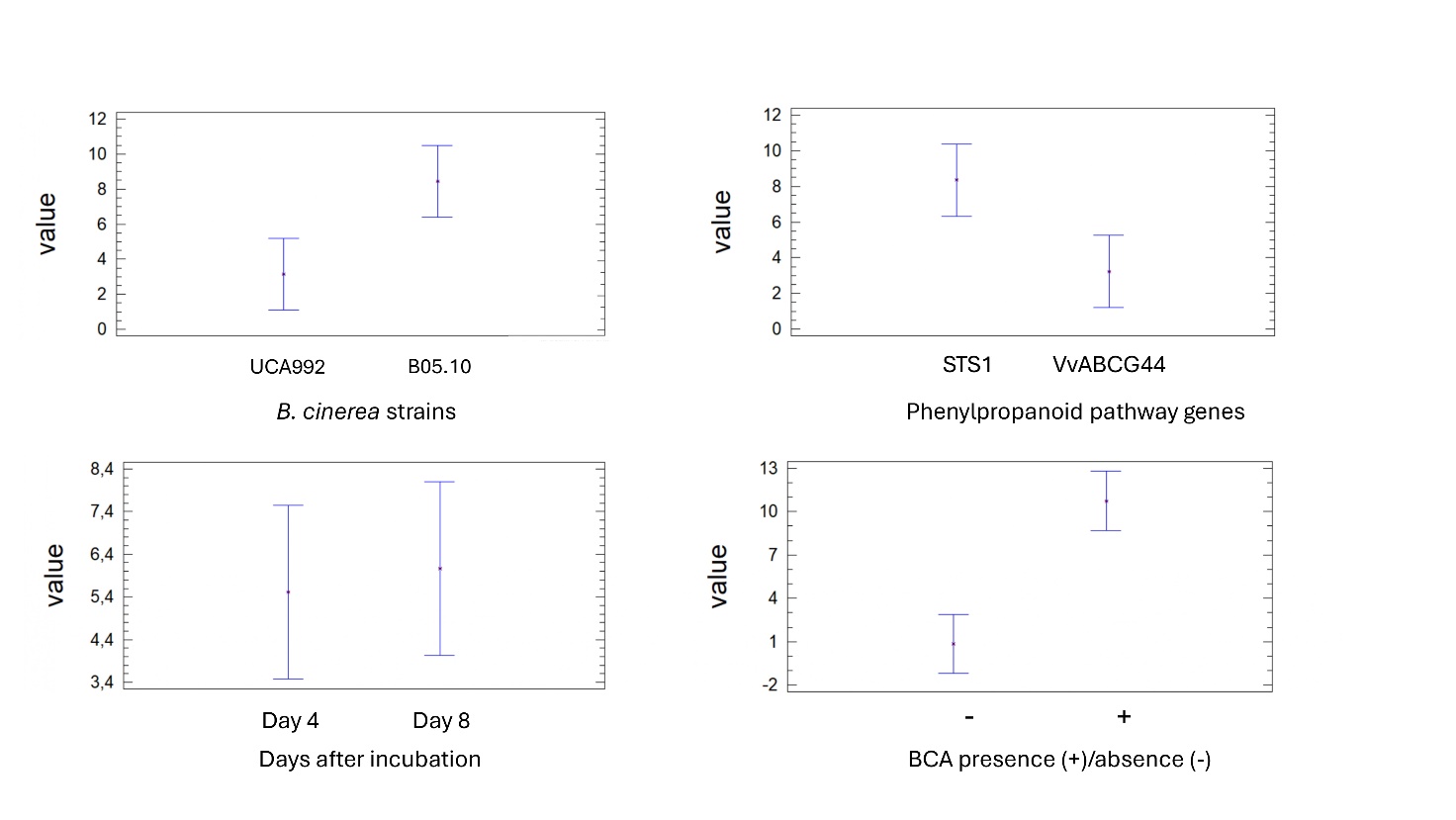


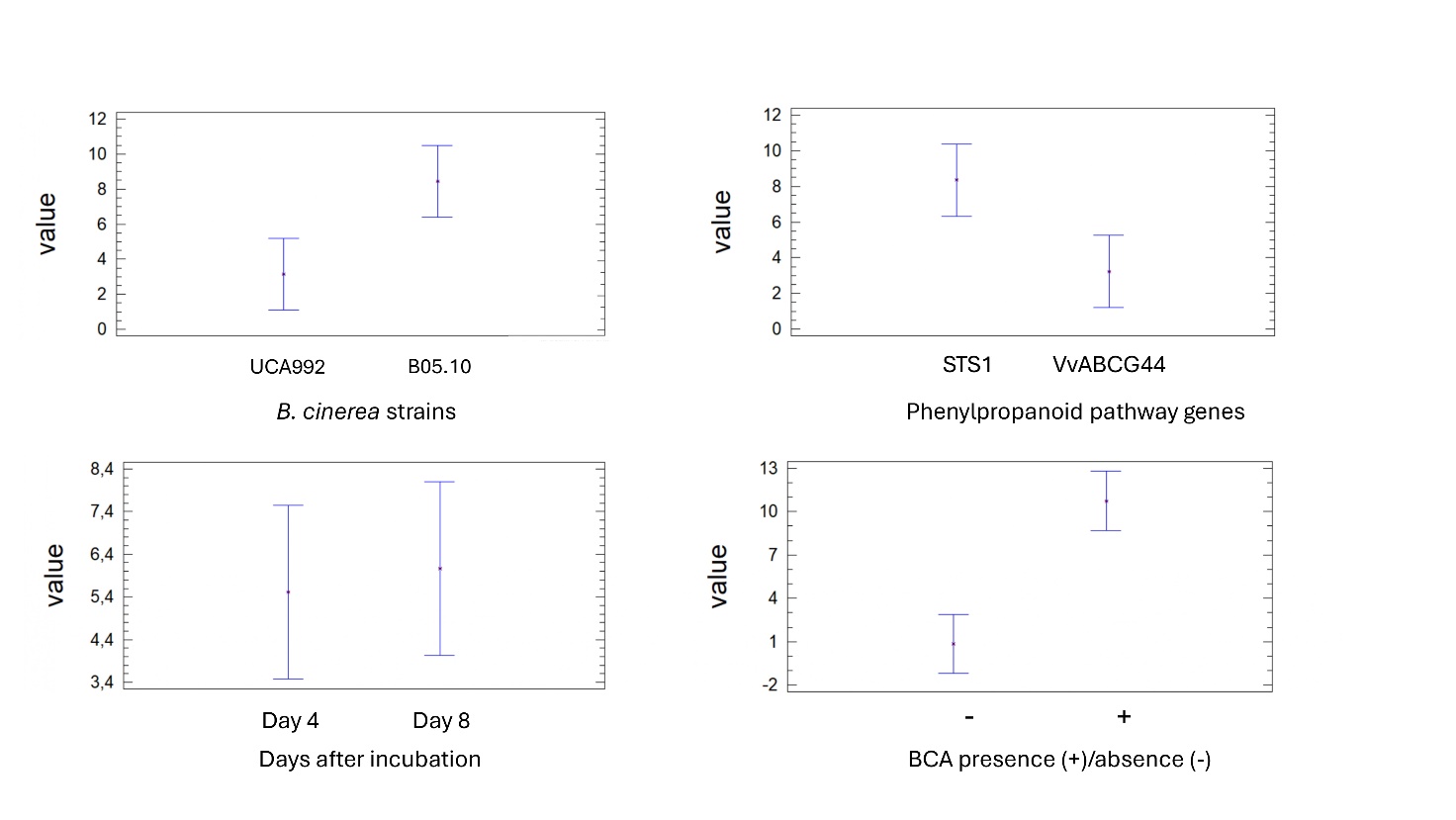

Supplement: Supplementary file 1 [file Table_1.docx]
